# Supplementary material for: Size-reduced DREADD derivatives for AAV-assisted multimodal chemogenetic control of neuronal activity and behavior
Source: Cell Rep Methods. 2024 Oct 21;4(10):100881. doi: 10.1016/j.crmeth.2024.100881 (PMC11573748; doi:10.1016/j.crmeth.2024.100881)
Supplement: Document S1. Figures S1–S5 [file mmc1.pdf]

Cell Reports Methods, Volume *4*

Supplemental information

Size-reduced DREADD derivatives for AAV-assisted multimodal chemogenetic control of neuronal activity and behavior

Takahito Miyake, Kaho Tanaka, Yutsuki Inoue, Yuji Nagai, Reo Nishimura, Takehito Seta, Shumpei Nakagawa, Ken-ichi Inoue, Emi Hasegawa, Takafumi Minamimoto, and Masao Doi

Supplementary Figure 1

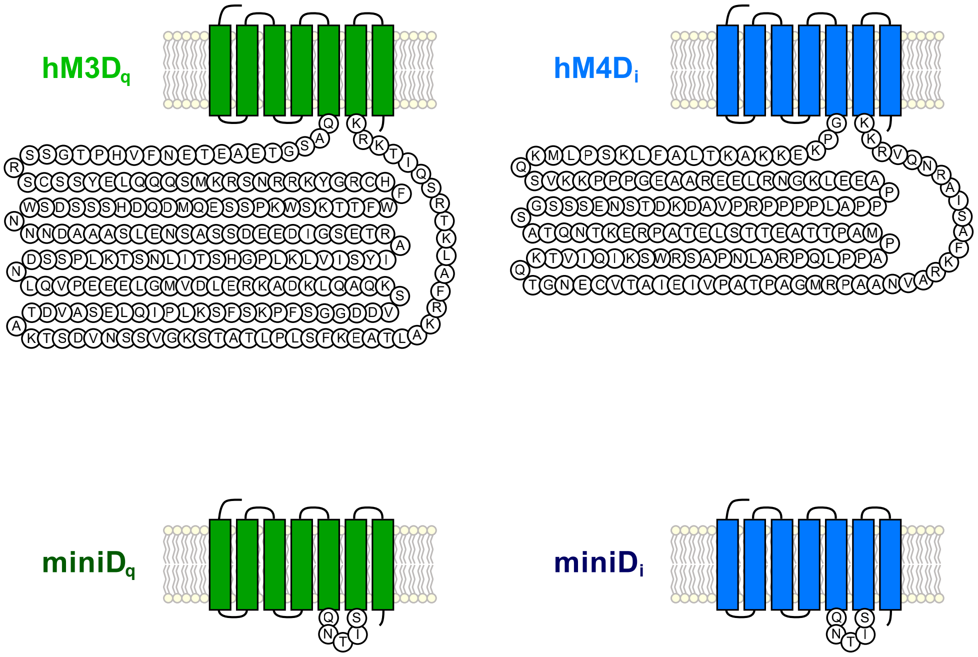

Supplementary Figure 1 (related to Fig. 1) | Snake plots showing the amino acid sequence of the ICL3 region of hM3D$_q$, hM4D$_i$, miniD$_q$ and miniD$_i$. We substituted the ICL3 of hM3D$_q$ and hM4D$_i$ with a 5-amino-acid peptide sequence Q-N-T-I-S.

<u>**Supplementary Figure 2**</u>

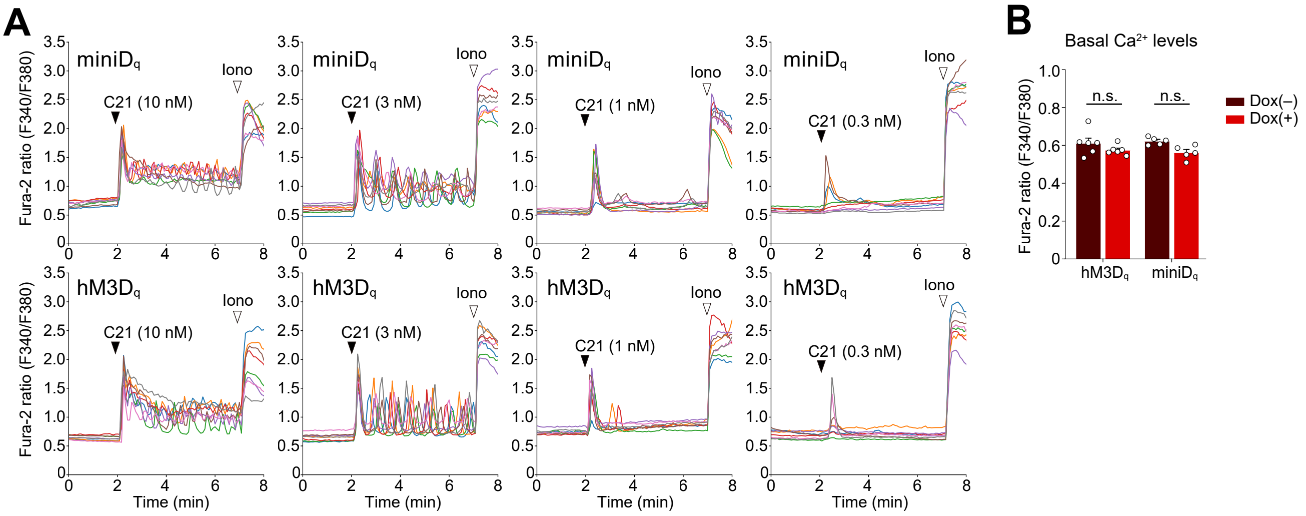

Supplementary Figure 2 (related to Fig. 2) | Fura-2 Ca^{2+} imaging of D_q-DREADD- expressing Flp-In TREx293 cells. (A) Representative traces in D_q-DREADD expressing cells. The traces of 10 nM C21-treated are the same as Figure 2D. Iono, ionomycin (3 µM). C21 evoked an immediate increase in Ca^{2+}, which is followed by oscillatory fluctuations of Ca^{2+}. Note that Ca^{2+} fluctuations after 3 nM C21 treatment were obvious and similar between hM3D_q and hM3D_q-ICL3$_{176}$ (miniD_q). **(B)** Comparison of basal Ca^{2+} levels between Dox-treated and -untreated cells for hM3D_q and miniD_q. n = 5−6 biological replicates. Cells were pretreated with Dox or vehicle for 18 h and monitored for $[Ca^{2+}]_i$ without C21 administration. Values are the means ± SEM. Two-way ANOVA followed by Sidak's multiple comparisons test. n.s., not significant.

Supplementary Figure 3

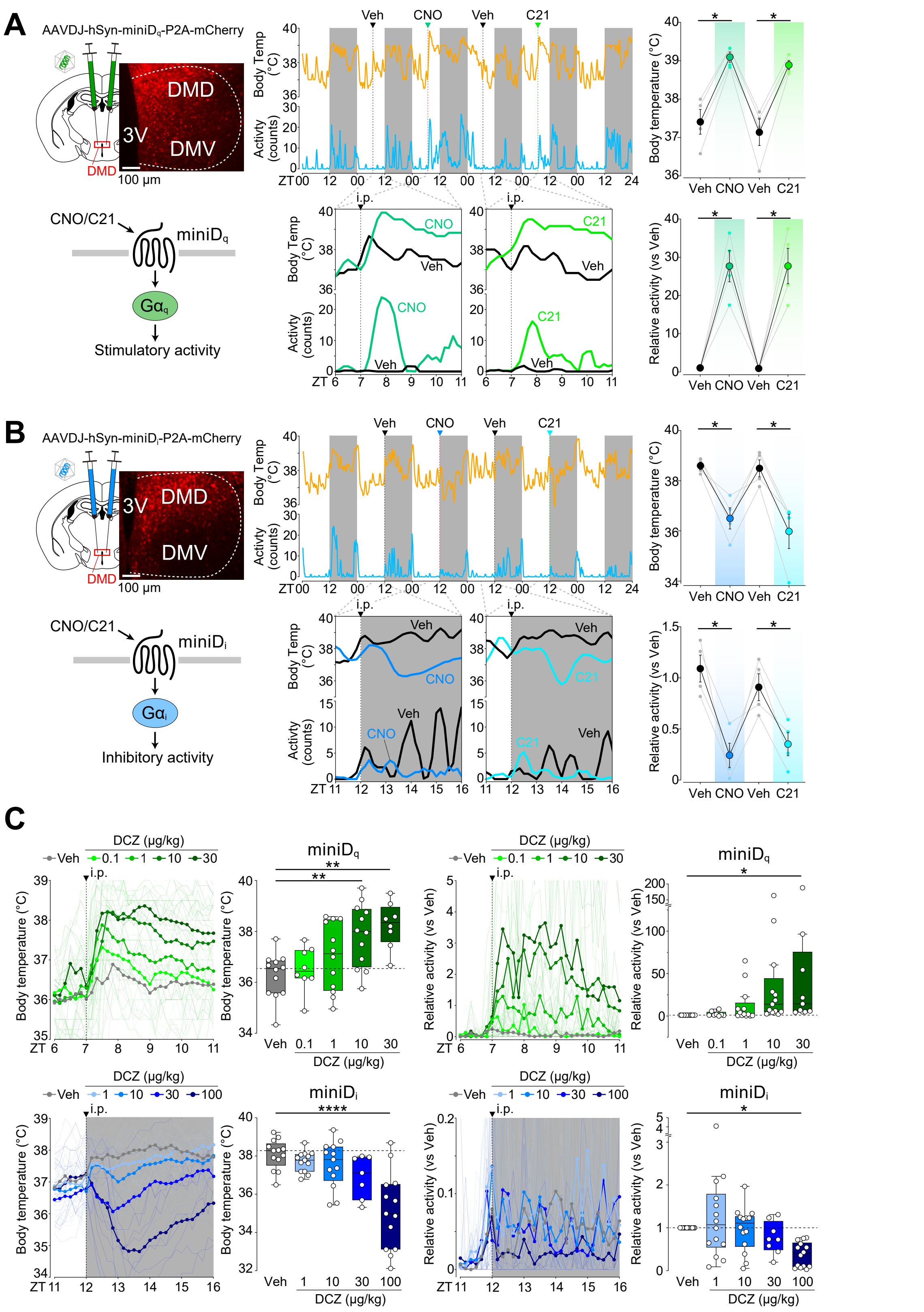

Supplementary Figure 3 (related to Fig. 4) | In vivo functionality of miniD$_i$/miniD$_q$-mediated neuronal activation or inhibition. (A) C21/CNO-induced upregulation of core body temperature and behavioral activity in mice virally expressing miniD$_q$ in the DMD. *n* = 4 mice. **(B)** C21/CNO-induced downregulation of core body temperature and behavioral activity in mice virally expressing miniD$_i$ in the DMD. *n* = 4 mice. Values in (A) and (B) are the mean ± SEM. **(C)** DCZ dose-dependent regulation of core body temperature and behavioral activity in mice virally expressing miniD$_q$ or miniD$_i$ in the DMD. *n* = 7–14 mice. In all experiments, we used male mice of 8-week-old. Mice were i.p. injected with DCZ (0.1–100 µg/kg), CNO (3 mg/kg), C21 (1 mg/kg), or vehicle (Veh, saline). *p < 0.05; **p < 0.01; ****p < 0.0001, one-way ANOVA followed by Tukey (A, B) or Dunnett (C) multiple comparisons test.

Supplementary Figure 4

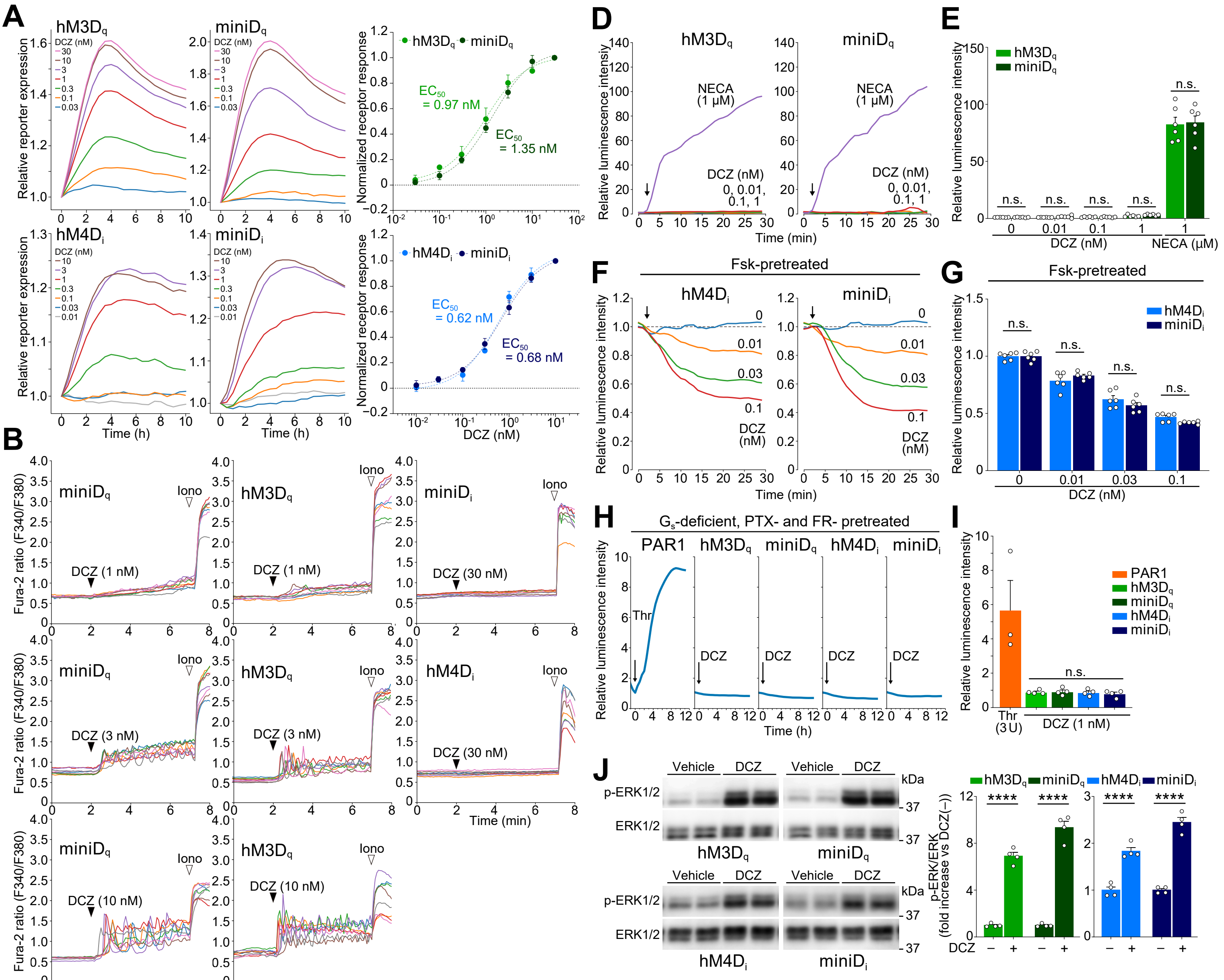

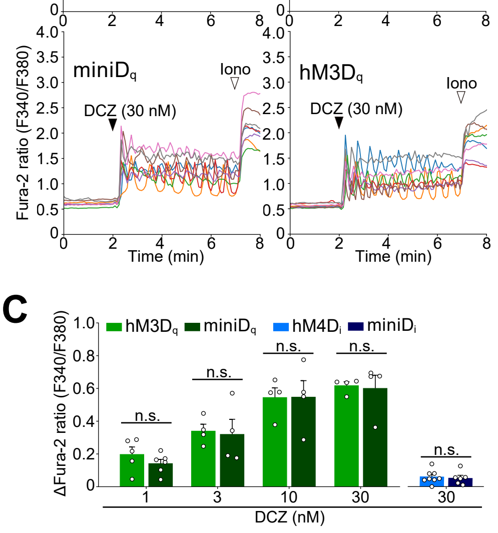

Supplementary Figure 4 (related to Fig. 5) | Conserved DCZ sensitivity in mini DREADDs in vitro. (A) Representative traces (left) and dose-response curves (right) of Tango reporter activity for hM3D$_q$, miniD$_q$, hM4D$_i$ and miniD$_i$ in response to DCZ treatment in HEK293 cells. *n* = 3−5 biological replicates. **(B)** Representative Fura-2 ratio traces in Flp-In TREx293 cells expressing hM3D$_q$, miniD$_q$, hM4D$_i$ or miniD$_i$. Arrowheads at 2 min represent the start of DCZ treatment. **(C)** Quantification of sustained Ca^{2+} levels in each DREADD receptor-expressing cells after treatment of increasing doses of DCZ. *n* = 4−8 biologically independent experiments. **(D–G)** Representative DCZ-induced changes in cAMP GloSensor reporter activity and their statistical quantification for hM3D$_q$ and miniD$_q$ (D, E) and hM4D$_i$ and miniD$_i$ (F, G). *n* = 6 biological replicates. **(H, I)** Representative SRF-RE reporter activity traces (H) and their statistical quantification (I). *n* = 3–4 biological replicates. Arrows, drug application. **(J)** Immunoblotting and densitometric analysis showing a significant and comparable increase in ERK phosphorylation after DCZ treatment in cells expressing hM3D$_q$, miniD$_q$, hM4D$_i$ and miniD$_i$. *n* = 4 biological replicates. Data were analyzed using two-way ANOVA followed by Sidak's multiple comparisons test (E, G, J, and hM3D$_q$ vs miniD$_q$ in C), unpaired Student's *t*-test (hM4D$_i$ vs miniD$_i$ in C) or one-way ANOVA followed by Tukey's multiple comaprisons test (I). Values are the means ± SEM. ****p < 0.0001; n.s., not significant.

<u>**Supplementary Figure 5**</u>

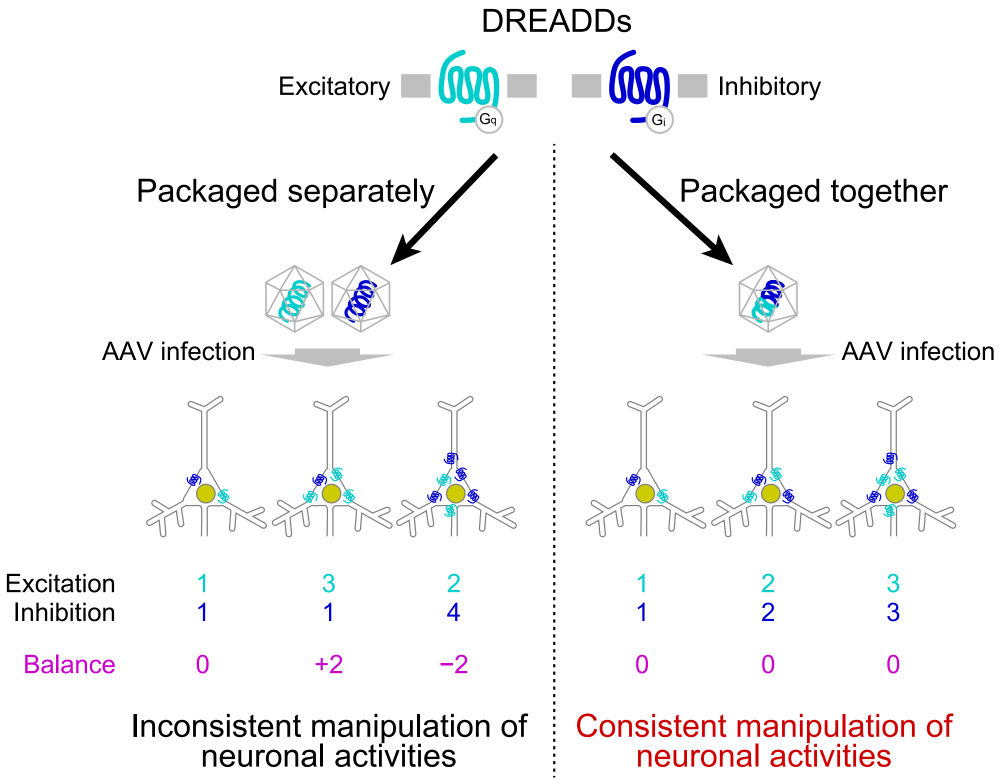

Supplementary Figure 5 (related to Figs. 4 and 5) | Schematic illustrating the potential advantage of co-packaging excitatory and inhibitory DREADDs in a single AAV capsid. When the two DREADDs are packaged separately in different AAV vectors, the ratio of excitatory to inhibitory DREADD expression can vary across neurons, resulting in inconsistent neuronal manipulation (left). In contrast, co-packaging the two DREADDs within a single capsid ensures the co-introduction of the two receptors to the same neurons with expression ratios equivalent between the two receptors, which would allow consistent up/down manipulation of targeted neuronal activities (right).
