# Supplementary material for: Size-reduced DREADD derivatives for AAV-assisted multimodal chemogenetic control of neuronal activity and behavior
Source: Cell Rep Methods. 2024 Oct 21;4(10):100881. doi: 10.1016/j.crmeth.2024.100881 (PMC11573748; doi:10.1016/j.crmeth.2024.100881)
Supplement: Document S2. Article plus supplemental information [file mmc2.pdf]

Cell Reports
Methods

Report

Size-reduced DREADD derivatives for AAV-assisted multimodal chemogenetic control of neuronal activity and behavior

Graphical abstract

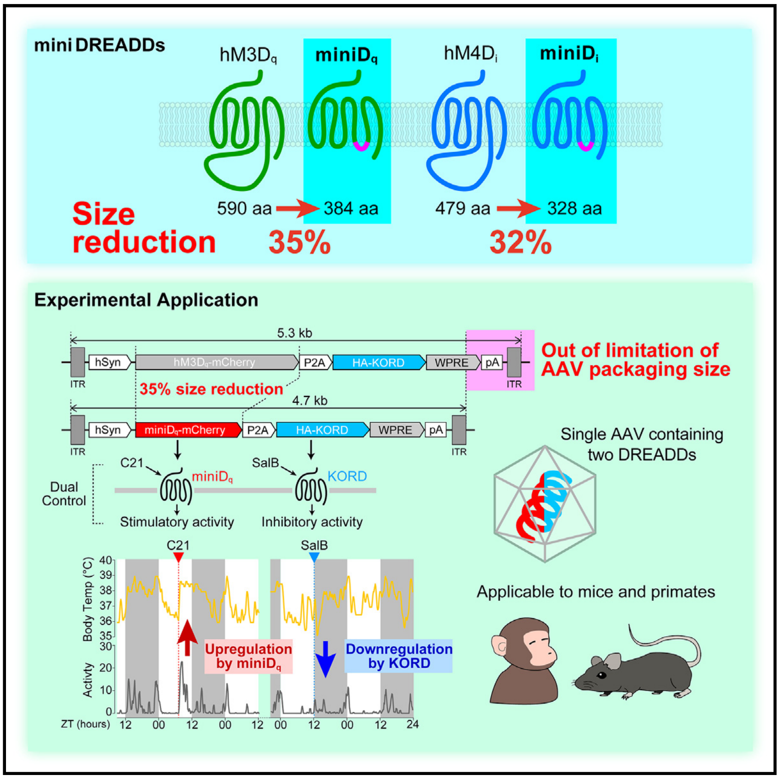

Highlights

- MiniD$_q$ and miniD$_i$ represent approximately 30% size-reduced hM3D$_q$ and hM4D$_i$, respectively
- MiniD$_q$ and miniD$_i$ possess the same receptor properties as hM3D$_q$ and hM4D$_i$, respectively
- The size reduction enables loading two DREADDs in a single AAV for bimodal regulation
- MiniD$_q$ and miniD$_i$ are useful for research in mice as well as non-human primates

Authors

Takahito Miyake, Kaho Tanaka, Yutsuki Inoue, ..., Emi Hasegawa, Takafumi Minamimoto, Masao Doi

Correspondence

tmiyake@pharm.kyoto-u.ac.jp (T.M.), doimasao@pharm.kyoto-u.ac.jp (M.D.)

In brief

Miyake et al. introduce miniaturized DREADD derivatives miniD$_q$ and miniD$_i$, which are approximately 30% smaller than hM3D$_q$ and hM4D$_i$, respectively. The miniaturized DREADD derivatives show preserved fundamental receptor functions, and their compact size enables the expression of miniD$_q$ and KORD via a single AAV vector for dual neuronal regulation.

Check for updates

Miyake et al., 2024, Cell Reports Methods *4*, 100881
October 21, 2024 © 2024 The Author(s). Published by Elsevier Inc.
https://doi.org/10.1016/j.crmeth.2024.100881

CellPress

Cell Reports Methods

CellPress
OPEN ACCESS

Report

Size-reduced DREADD derivatives for AAV-assisted multimodal chemogenetic control of neuronal activity and behavior

Takahito Miyake,[1,4,*] Kaho Tanaka,[1,4] Yutsuki Inoue,[1,4] Yuji Nagai,[2] Reo Nishimura,[1] Takehito Seta,[1] Shumpei Nakagawa,[1] Ken-ichi Inoue,[3] Emi Hasegawa,[1] Takafumi Minamimoto,[2] and Masao Doi[1,5,*]
[1]Department of Systems Biology, Graduate School of Pharmaceutical Sciences, Kyoto University, Sakyō-ku, Kyoto 606-8501, Japan
[2]Advanced Neuroimaging Center, National Institutes for Quantum Science and Technology, Chiba 263-8555, Japan
[3]Systems Neuroscience Section, Center for the Evolutionary Origins of Human Behavior, Kyoto University, Inuyama 484-8506, Japan
[4]These authors contributed equally
[5]Lead contact
*Correspondence: tmiyake@pharm.kyoto-u.ac.jp (T.M.), doimasao@pharm.kyoto-u.ac.jp (M.D.)
https://doi.org/10.1016/j.crmeth.2024.100881

MOTIVATION The limited packaging capacity of the adeno-associated virus (AAV) makes it unfeasible to accommodate multiple gene tools within a single vector. Therefore, success in decreasing the size of genetic tools has had a profound impact on biomedical research. However, the downsizing of designer receptors exclusively activated by designer drugs (DREADDs), a crucial tool for modulating neural functions, remains an unfilled opportunity. In this study, we address this gap by reducing the size of the widely utilized DREADD subtypes, hM3D$_q$ and hM4D$_i$, by approximately 30%. Our miniaturized DREADDs facilitate bimodal regulation of neuronal activity—enabling both excitation and inhibition—using a single AAV vector, thereby offering a versatile and compact chemogenetic platform.

SUMMARY

Designer receptors exclusively activated by designer drugs (DREADDs) are engineered G-protein-coupled receptors that afford reversible manipulation of neuronal activity *in vivo*. Here, we introduce size-reduced DREADD derivatives miniD$_q$ and miniD$_i$, which inherit the basic receptor properties from the G$_q$-coupled excitatory receptor hM3D$_q$ and the G$_i$-coupled inhibitory receptor hM4D$_i$, respectively, while being approximately 30% smaller in size. Taking advantage of the compact size of the receptors, we generated an adeno-associated virus (AAV) vector carrying both miniD$_q$ and the other DREADD family receptor (κ-opioid receptor-based inhibitory DREADD [KORD]) within the maximum AAV capacity (4.7 kb), allowing us to modulate neuronal activity and animal behavior in both excitatory and inhibitory directions using a single viral vector. We confirmed that expressing miniD$_q$, but not miniD$_i$, allowed activation of striatum activity in the cynomolgus monkey (*Macaca fascicularis*). The compact DREADDs may thus widen the opportunity for multiplexed interrogation and/or intervention in neuronal regulation in mice and non-human primates.

INTRODUCTION

Multiplexed dissection of neural circuitry and behavior is crucial to investigate complex brain functions for health and diseases. Adeno-associated virus (AAV) is the most promising vector for this purpose, as it enables highly efficient, nontoxic, stable long-term transgene expression in neurons.[1–3] However, the limited capacity of AAV to package DNA (less than 4.7 kb) makes it unfeasible to accommodate multiple gene tools (or elements exceeding 4.7 kb) within a single vector.[4–8] Therefore, the success in decreasing the size of useful DNA tools has had a profound impact on biomedical research utilizing AAV (see examples such as CRISPR-Cas9[9] and base-editing tools[10,11]). However, despite these efforts, reducing the size of designer receptors exclusively activated by designer drugs (DREADDs) is still an unfilled opportunity for neuroscience. For example, in experiments where two DREADD tools need to be introduced simultaneously for bimodal regulation of neuronal activity, researchers currently perform separate experiments in which either an excitatory or inhibitory receptor is expressed because current DREADD sizes cannot permit double loading on a single AAV capsid.[12] If a size-reduced DREADD derivative(s) becomes available, this enables its use in conjunction with other chemogenetic tools in a single AAV capsid, thereby facilitating

© 2024 The Author(s). Published by Elsevier Inc.
This is an open access article under the CC BY license (http://creativecommons.org/licenses/by/4.0/).

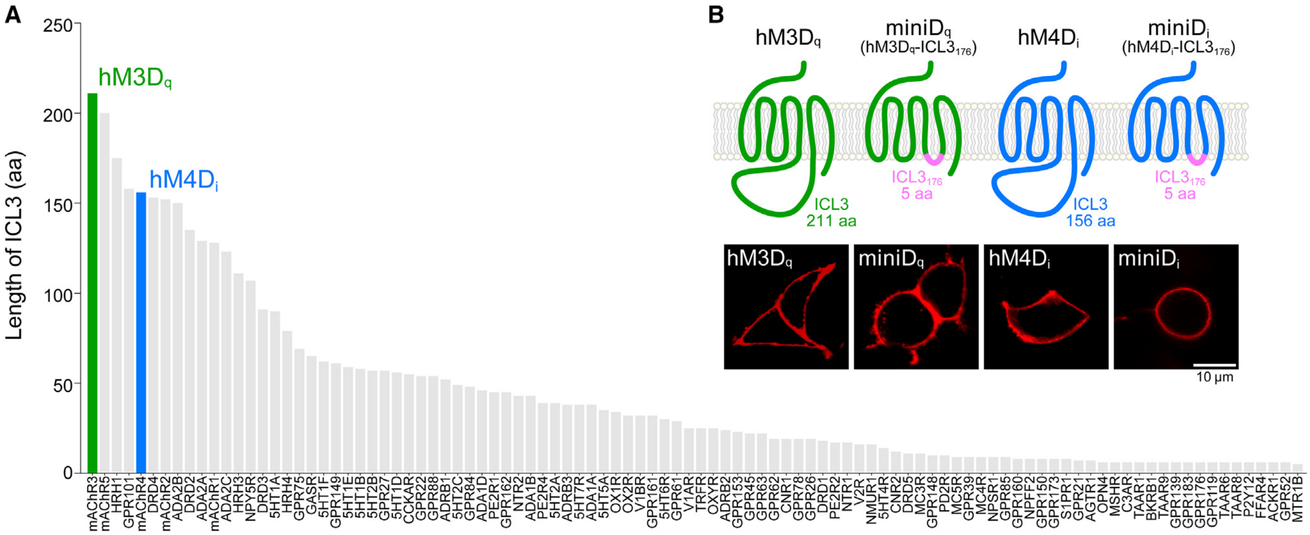

Figure 1. DREADDs with size-reduced ICL3s

(A) Human class A GPCRs rank ordered for ICL3 length. Top 100 receptors are shown.

(B) Schematic snake plot representation of $hM3D_q$, $miniD_q$, $hM4D_i$, and $miniD_i$ and their subcellular expression in HEK293 cells. mCherry was fused to each receptor for visualization. Snake plots showing the ICL3 sequence of $hM3D_q$, $miniD_q$, $hM4D_i$, and $miniD_i$ are available in Figure S1.

bidirectional interrogations (i.e., excitation and inhibition) of targeted cell circuitry by ensuring the co-introduction of the two tools into the same composite neurons. Attempts to produce size-reduced DREADD tools, however, have not been reported so far.

Here, we report obtaining downsized DREADD derivatives, $miniD_q$ and $miniD_i$, both characterized by a shortened length of the third intracellular loop (ICL3) compared to $hM3D_q$ and $hM4D_i$, respectively. We verified their basic receptor characteristics and explored their applications, including their bimodal regulation of neuronal activity using a single vector.

RESULTS

Shortening ICL3 does not affect plasma membrane expression of DREADDs

Human class A G-protein-coupled receptors (GPCRs), rank ordered for ICL3 length, characterized that the muscarinic acetylcholine receptors 3 and 4 (mAChR3 and mAChR4), the ancestors of human mAChR-based $hM3D_q$ and $hM4D_i$, possess the longest and fifth longest ICL3s among 312 class A members in GPCRdb[13] (Figure 1A). mAChR3 (and its mutant $hM3D_q$) possesses an ICL3 of 211 amino acids, accounting for ~36% of its total length (Figure 1B); mAChR4 (and its mutant $hM4D_i$) possesses an ICL3 of 156 amino acids, approximating ~33% of the whole protein length[14] (Figure 1B). We substituted the ICL3s of $hM3D_q$ and $hM4D_i$ with a 5-amino-acid peptide sequence, Q-N-T-I-S, which corresponds to the hGpr176 ICL3 sequence devoid of a proline residue at its N terminus (Figure 1B, $ICL3_{176}$; see also Figure S1).[15–18] Prior to beginning functional assays, we asked whether this ICL3 substitution might affect subcellular expression of the receptors. Confocal microscopy revealed that all the receptors tested, $hM3D_q$, $hM4D_i$, and their respective mutants, $hM3D_q$-$ICL3_{176}$ and $hM4D_i$-$ICL3_{176}$, both being smaller than the ancestral receptors in size due to the reduction of the ICL3, were similarly located in the plasma membrane when expressed in HEK293 cells (Figure 1B), thus suggesting no deleterious effect on protein production or the cell surface location of the receptors due to the introduction of the $ICL3_{176}$.

Conserved ligand selectivity and sensitivity of $hM3D_q$-$ICL3_{176}$ and $hM4D_i$-$ICL3_{176}$

The replacement of the ICL3, however, may cause an alteration in, for example, cognate ligand sensitivity of $hM3D_q$ and/or $hM4D_i$. To test this possibility, we performed a β-arrestin recruitment assay[19] (Figure 2A). HEK293 cells were transfected with a DREADD-Tango of interest and treated with different concentrations of the cognate ligand compound C21. In accordance with a standard PREST-Tango method,[19] a C-terminal tail of the V2 vasopressin receptor (V2 tail), the tobacco etch virus protease (TEV)-cleavage site, and the tetracycline transactivator (tTA) were fused in tandem with the C terminus of the receptors. The β-arrestin2-TEV fusion protein and tTA-responsive luciferase reporter were used (Figure 2A). All the receptors tested showed a concentration-dependent response to C21 (Figure 2B, left), with similar EC_{50} values between $hM3D_q$ and $hM3D_q$-$ICL3_{176}$ and between $hM4D_i$ and $hM4D_i$-$ICL3_{176}$ (Figure 2B, right), indicating the undisturbed ligand sensitivity of $hM3D_q$-$ICL3_{176}$ and $hM4D_i$-$ICL3_{176}$ (note that we rename $hM3D_q$-$ICL3_{176}$ and $hM4D_i$-$ICL3_{176}$ as $miniD_q$ and $miniD_i$, respectively, in a later section). We found little or no discernable response of the receptors toward ACh. We used up to 100 μM of ACh, a concentration higher than the physiological peak levels of ACh release in the brain (~2 μM)[20]; however, as expected, $hM3D_q$ and $hM4D_i$ did not respond to this treatment,[21,22] and this

CellPress
OPEN ACCESS

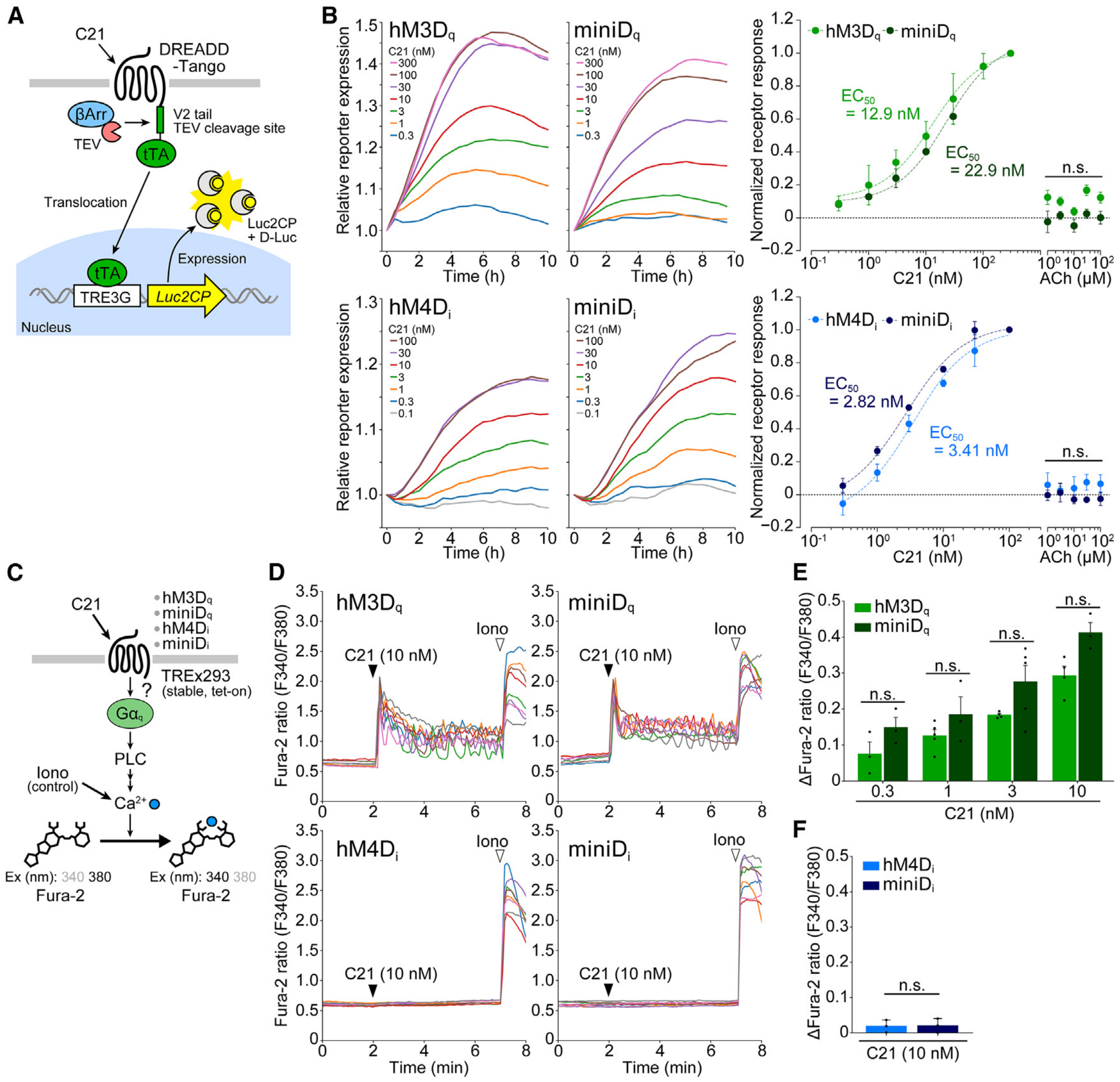

Figure 2. Conserved ligand response and downstream Ca^{2+} signaling direction by mini DREADDs

(A) Schematic of β-arrestin recruitment Tango assay.

(B) Representative traces (left) and dose-response curves (right) of Tango reporter activity for $hM3D_q$, $miniD_q$, $hM4D_i$, and $miniD_i$ in response to C21 treatment in HEK293 cells. n = 3–5 biological replicates.

(C) Schematic of Fura-2-based ratiometric intracellular Ca^{2+} imaging.

(D) Representative Fura-2 ratio traces in Flp-In TREx293 cells expressing $hM3D_q$, $miniD_q$, $hM4D_i$, and $miniD_i$. Arrowheads at 2 min represent the start of C21 treatment. Ionomycin (Iono; 3 μM) was applied post hoc for the validation of imaging and cell viability.

(E) Quantification of sustained Ca^{2+} levels in $hM3D_q$- or $miniD_q$-expressing cells after treatment with increasing doses of C21. Raw Fura-2 traces are available in Figure S2A. n = 3–5 biological replicates.

(F) Quantification of Ca^{2+} levels for $hM4D_i$ and $miniD_i$ after treatment with C21 (10 nM). n = 3.

Data were analyzed using two-way ANOVA followed by Sidak's multiple comparisons test (B and E) or unpaired Student's t test (F). Values are the means ± SEM. n.s., not significant.

independence (or unresponsiveness to ACh) was also observed for $hM3D_q$-$ICL3_{176}$ and $hM4D_i$-$ICL3_{176}$ (Figure 2B, right), verifying the unperturbed ligand selectivity of the receptors.

Conserved downstream activity of $hM3D_q$-$ICL3_{176}$ and $hM4D_i$-$ICL3_{176}$

Next, we monitored downstream signaling selectivity. In the following experiments, we used Flp-In TREx293 cells (tet-on HEK293 cells) expressing $hM3D_q$, $hM4D_i$, $hM3D_q$-$ICL3_{176}$, or $hM4D_i$-$ICL3_{176}$ (Figures 2 and 3). Unless otherwise mentioned, cells were treated with doxycycline (Dox) prior to experiments. Upon C21 treatment, cells expressing $hM3D_q$ displayed expected intracellular calcium concentration increases in a dose-dependent manner, as determined by Fura-2AM fluorometry (Figures 2C–2E and S2A). A similar dose dependency was observed for $hM3D_q$-$ICL3_{176}$ (no significant difference between

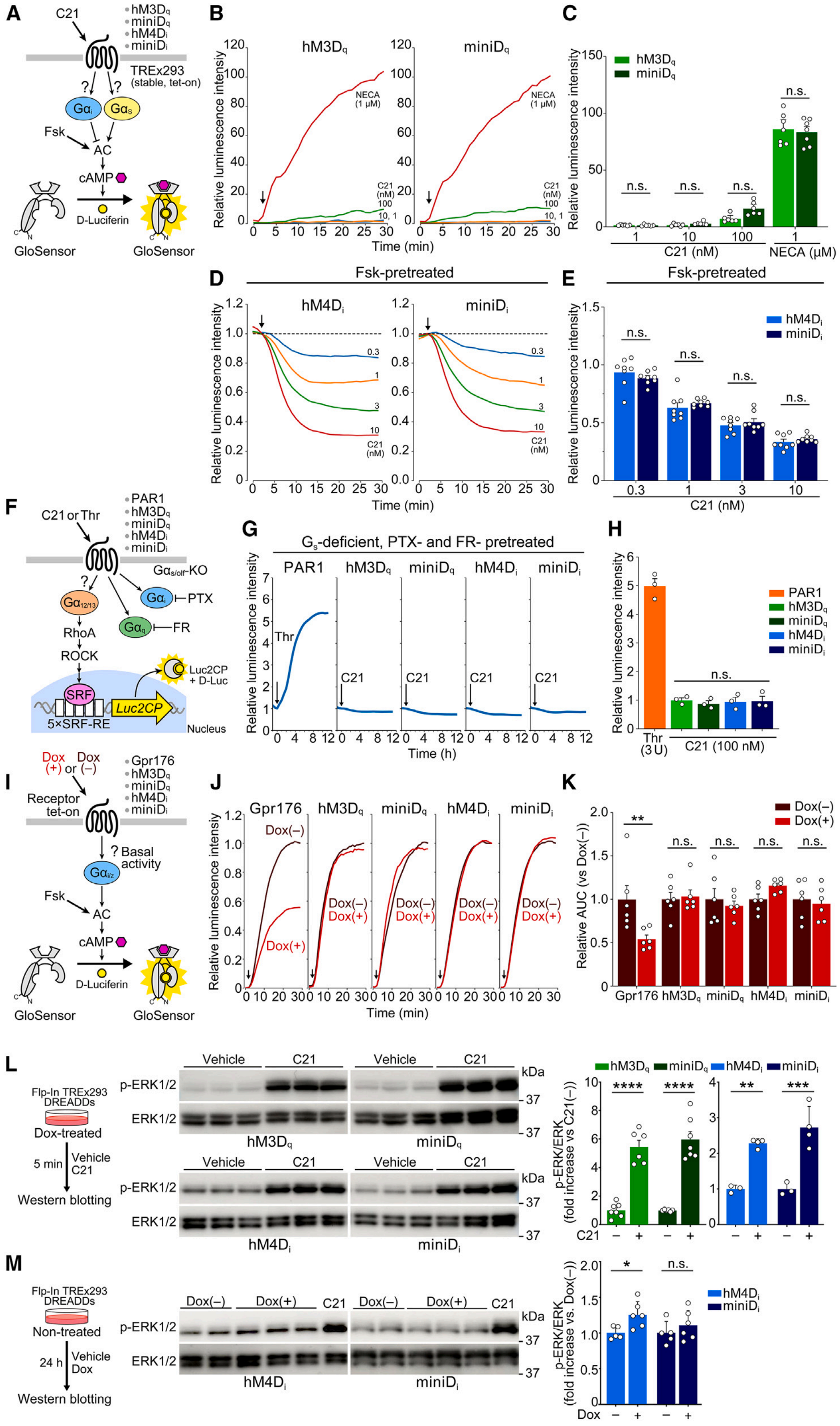

(legend on next page)

$hM3D_q$ and $hM3D_q$-$ICL3_{176}$ at any doses tested, Figure 2E). An immediate increase in $[Ca^{2+}]_i$, caused by C21, was followed by sustained, oscillatory fluctuations of $[Ca^{2+}]_i$, a phenomenon typical for G_q-coupled signaling,[23,24] in both $hM3D_q$ and $hM3D_q$-$ICL3_{176}$ (Figure S2A; see, e.g., 3 nM C21), further indicating preserved Ca^{2+} control by these receptors. Not surprisingly, no detectable Ca^{2+} response was observed for cells expressing $hM4D_i$, which is designed to couple to G_i (Figures 2D and 2F).[21] $hM4D_i$-$ICL3_{176}$-expressing cells did not exhibit a Ca^{2+} increase, either (Figures 2D and 2F), verifying that there is no gain-of-function G_q/Ca^{2+} activity of $hM4D_i$-$ICL3_{176}$. A strong increase in $[Ca^{2+}]_i$ after ionomycin (Iono) treatment (Figure 2D) confirmed cell viability for all tested cells.

We next searched for a stimulatory or inhibitory activity on cAMP signaling. In agreement with selective G_q coupling, cells expressing $hM3D_q$ or $hM3D_q$-$ICL3_{176}$ did not show increased cAMP accumulation after stimulation by C21, which contrasts with 5′-N-ethylcarboxamido-adenosine (NECA), an agonist for the endogenous adenosine 2B receptor present in the cells, evoking pronounced cAMP accumulation (Figures 3A–3C; adenosine 2B receptor couples to G_s[25]). In experiments examining the inhibitory response of cAMP for G_i-linked $hM4D_i$ (and its mutant $hM4D_i$-$ICL3_{176}$), cells received forskolin (Fsk), a cAMP enhancer, before C21 application (Figures 3A, 3D, and 3E). cAMP levels were decreased immediately after the C21 treatment in cells expressing $hM4D_i$ (Figures 3D and 3E). cAMP levels in $hM4D_i$-$ICL3_{176}$-expressing cells were also decreased after the treatment in a C21 concentration-dependent manner that was statistically indistinguishable from that observed in the original $hM4D_i$-expressing cells (Figures 3D and 3E), which provides support for the intact G_i signaling mediated by $hM4D_i$-$ICL3_{176}$.

Additionally, we studied G_{12}/G_{13}-mediated luciferase reporter gene expression utilizing the serum response factor response element (SRF-RE) as reported.[26,27] To ensure the specificity, we performed this assay using $G\alpha_s/G\alpha_{olf}$-deficient TREx293 cells (*GNAS*$^{-/-}$;*GNAL*$^{-/-}$) in the presence of pertussis toxin (PTX) for the inhibition of G_i and FR900359 (FR) for the inhibition of G_q (Figure 3F). As expected, we observed ligand-dependent upregulation of the G_{12}/G_{13}-coupled thrombin (Thr) receptor PAR1 (protease-activated receptor 1). Under these conditions, there was no detectable upregulation of the reporter in our cells after C21 treatment, indicating that $hM3D_q$-$ICL3_{176}$ and $hM4D_i$-$ICL3_{176}$ were both comparable to $hM3D_q$ and $hM4D_i$, with no appreciable gain of function to $G_{12/13}$ activity (Figures 3G and 3H).

The $ICL3_{176}$ sequence was obtained from Gpr176, a constitutively active GPCR possessing cAMP inhibitory activity[15]; thus, we checked the possible basal activity of the receptors. To do this, we leveraged the tet-inducible-receptor-expressing cells, and Fsk-induced cAMP accumulation was compared between Dox-treated and untreated cells without C21 treatment (Figure 3I). Dox did not substantially affect the cAMP accumulation profiles in any of the tested cells except for Gpr176-expressing cells, which produced significant attenuation of cAMP accumulation following Dox treatment ($p < 0.01$ vs. untreated, Figures 3J and 3K). In parallel, we also checked $[Ca^{2+}]_i$ levels and found that Dox induction of $hM3D_q$ or $hM3D_q$-$ICL3_{176}$ had no appreciable effect on basal $[Ca^{2+}]_i$ monitored via Fura-2 (Figure S2B).

Based on these observations thus far (Figures 1, 2, and 3), we accordingly renamed $hM3D_q$-$ICL3_{176}$ and $hM4D_i$-$ICL3_{176}$ as $miniD_q$ and $miniD_i$, respectively, as they retain G protein selectivity and ligand specificity after being reduced in size.

Comparison between original DREADDs and $miniD_i$/$miniD_q$ in ERK phosphorylation

In addition to G-protein-dependent signaling, DREADDs also activate the extracellular signal-regulated kinase (ERK) 1/2 pathway independently of receptor coupling to G proteins.[28,29] C21-induced ERK phosphorylation was comparable between $miniD_q$ and $hM3D_q$ and between $miniD_i$ and $hM4D_i$ (Figure 3L). However, we noticed a slight difference in basal ERK phosphorylation between $hM4D_i$-expressing cells and $miniD_i$-expressing cells (Figure 3L, see lanes for C21-untreated cell samples). More specifically, $hM4D_i$ appeared to have a slightly higher constitutive activity for ERK phosphorylation than $miniD_i$. To verify this difference, we compared basal ERK phosphorylation

Figure 3. Comparable modulation of cAMP and ERK signaling by original and mini DREADDs

(A) Schematic representation of GloSensor reporter assay for evaluation of G_s- or G_i-mediated cAMP signaling. To monitor G_i activity, cells were pretreated with forskolin (Fsk).

(B−E) Representative C21-induced changes in cAMP GloSensor reporter activity and their statistical quantification for $hM3D_q$ and $miniD_q$ (B and C) and $hM4D_i$ and $miniD_i$ (D and E). n = 6 biological replicates. Arrows, C21 or NECA application.

(F) Schematic representation of SRF-RE reporter assay for evaluating $G_{12/13}$-based signaling. PAR1 was used as a positive control. $G_{s/olf}$-deficient cells were pretreated with PTX and FR.

(G and H) Representative SRF-RE reporter activity traces (G) and their statistical quantification (H). n = 3 biological replicates. Thr, thrombin.

(I) Schematic design for evaluation of receptor basal activity. Gpr176 was used as a positive control. Agonist-independent, basal inhibitory activity for Fsk-induced cAMP accumulation was evaluated using Flp-In TREx293 doxycycline (Dox)-inducible receptor-expressing cells; cells received Dox or vehicle 18 h before experiments.

(J) Representative cAMP GloSensor activity traces in Dox-treated (+) and untreated (−) cells. Arrows indicate the start of Fsk treatment. Attenuated cAMP accumulation was only observed for Gpr176 Dox (+) cells.

(K) Quantification of area under the curve (AUC) in (J). n = 5–8 biological replicates.

(L) Immunoblotting and densitometric analysis showing a significant and comparable increase in ERK phosphorylation after C21 treatment in cells expressing $hM3D_q$, $miniD_q$, $hM4D_i$, and $miniD_i$. n = 3–7 biological replicates.

(M) Immunoblots and densitometric analysis showing an agonist-independent basal expression of phospho-ERK in $hM4D_i$-induced cells but not $miniD_i$-induced cells. Cells were either treated or untreated with Dox without C21. C21-treated cell lysate samples were loaded in parallel as a control. n = 5–6 biological replicates.

Data were analyzed using two-way ANOVA followed by Sidak's multiple comparisons test (C, E, and K–M) or one-way ANOVA followed by Tukey's multiple comparisons test (H). Values are the mean ± SEM. ****$p < 0.0001$, ***$p < 0.001$, **$p < 0.01$, *$p < 0.05$, and n.s., not significant.

in cells with and without receptor expression (Dox(+) vs. Dox(−)). A significant increase in basal ERK phosphorylation was observed when hM4D$_i$ was expressed but not when miniD$_i$ was expressed ($p < 0.05$, only for hM4D$_i$-Dox(+) vs. Dox(−); Figure 3M), indicating reduced constitutive activity at ERK signaling for miniD$_i$.

Application of miniD$_q$ and miniD$_i$ to neuronal modulation *in vivo*

To test the applicability of miniD$_q$ and miniD$_i$ *in vivo*, we injected AAV expressing miniD$_q$ or miniD$_i$ in the dorsal part of the dorsomedial hypothalamus (DMD), a brain region involved in controlling body temperature and activity-induced thermogenesis.[30] As shown in Figures S3A and S3B, activation of the neurons in the DMD in miniD$_q$-infected mice resulted in a significant increase in body temperature and behavioral activity while inhibiting them in miniD$_i$-infected mice, in contrast, lowered body temperature and locomotor activity. In these experiments, we confirmed that both miniD$_q$ and miniD$_i$ were able to be activated by C21 as well as CNO, the two major drugs[22,31,32] for DREADD activation *in vivo*. Drugs were applied to mice in the middle of resting phase (zeitgeber time [ZT]07, Figure S3A) or in the beginning of active phase (ZT12, Figure S3B) when body temperature and locomotor activity were lowest or highest, respectively (ZT00 denotes lights on and ZT12 lights off). Vehicle treatment between drug applications did not induce corresponding phenomena (Figures S3A and S3B). We further verified that the magnitude of the drug-induced changes in body temperature and locomotor activity was increased in a dose-dependent manner (Figure S3C).

Having observed the applicability of mini DREADD *in vivo*, we finally sought to exploit the potential benefit that could be offered from the size-reduced derivative of DREADDs. The limited capacity of AAV to accommodate foreign DNA (<4.7 kb)[33] motivated us to apply mini DREADDs to develop a virus carrying multiple tools simultaneously, a task sometimes required for neurosciences. As an example, we generated an AAV carrying both miniD$_q$ and the κ-opioid receptor-based inhibitory DREADD (KORD)[34] with a length of 4.7 kb, the limit of the AAV's capacity (Figure 4A), which cannot be accomplished with the combination of hM3D$_q$ and KORD, which total ∼5.3 kb. The self-cleaving 2A sequence was inserted between miniD$_q$ and KORD to achieve bicistronic gene expression. These two receptors respond to mutually independent chemical agonists, C21 for miniD$_q$ and salvinorin B (SalB) for KORD,[34] thus enabling multiplexed chemogenetic study. Primary neuronal cells were infected with the viral vector AAV-hSyn-miniD$_q$-P2A-KORD. Immunofluorescence confirmed the co-expression of miniD$_q$ and KORD in the same cells (Figure 4B). *In vitro* Ca^{2+} imaging further demonstrated that neurons that were able to be activated by miniD$_q$ were also consistently inhibited by KORD (Figure 4B), indicating functional co-expression of the two receptors in the same cells. Therefore, our system developed here enabled us to bidirectionally modulate target cell activity with single vector infection. To use this system *in vivo*, we applied it to the DMD (Figure 4C). Administration of C21, but not vehicle, at ZT07 led to a significant increase in body temperature and locomotor activity ($p < 0.01$ for body temperature; $p < 0.05$ for locomotor activity, vehicle vs. C21, Figure 4C). Importantly, in the same mice, SalB administered at ZT12, but not control vehicle treatment, caused a significant decrease in body temperature and locomotor activity ($p < 0.01$ for both parameters, vehicle vs. SalB, Figure 4C), demonstrating the bidirectional modulation of DMD function *in vivo* with our system. Precise viral infection and the resulting co-expression of miniD$_q$ and KORD in the DMD were finally verified by post hoc immunohistochemistry (IHC) (Figure 4C).

Potential application to non-human primates

To begin to address the question of applicability of our miniD$_q$/D$_i$ to monkey, AAV viruses encoding either miniD$_q$, miniD$_i$, hM3D$_q$, or hM4D$_i$ were stereotaxically injected in parallel to four comparable regions in the striatum of the cynomolgus monkey (see Figure 5A). Nearly equal expression of the receptors was verified via [^{11}C]DCZ positron emission tomography (PET) imaging, a method used to see *in vivo/in situ* binding of DCZ (deschloroclozapine), a widely used actuator for hM3/hM4 DREADDs in monkeys[35–39] (Figure 5B). Increased [^{18}F]-fluoro-deoxy-glucose ([^{18}F]FDG) uptake, reflecting neuronal activation, was observed in the region expressing hM3D$_q$ but not hM4D$_i$ after intravenous administration of DCZ relative to vehicle control (Figure 5C), which was recapitulated by the miniD$_q$-mediated, but not miniD$_i$-mediated, increased accumulation of [^{18}F]FDG observed after the administration of DCZ (Figure 5C). Finally, we confirmed that DCZ acts as an actuator for miniD$_i$ and miniD$_q$ using the Tango assay, Fura-2 Ca^{2+} imaging, GloSensor cAMP assay, and ERK phosphorylation assay (see Figure S4).

DISCUSSION

In this study, we obtained size-reduced DREADD derivatives, miniD$_q$ and miniD$_i$, which inherit the basic receptor characteristics of hM3D$_q$ and hM4D$_i$ while being smaller in size by ∼30%. We then exemplified the potential size merit(s) by showing the generation of AAV carrying miniD$_q$ and KORD simultaneously within the length of the maximum AAV capacity. This ensured the co-expression of the two independent tools in the same cells, a condition not readily attained by double infection of separate AAVs. The 2A sequence between miniD$_q$ and KORD also ensured almost equivalent expression of the two receptors across cell types and mice tested, a feature that would also help to increase the reproducibility of data by reducing the potential variation from multi-tool infection. We found equivalent co-expression of miniD$_q$ and KORD in the same neuronal population of DMD after infection, and reflecting this, all mice subjected to the AAV-miniD$_q$-P2A-KORD virus exhibit consistent but opposing activity responses after treatment of C21 and SalB in the same test mice. The use of the size-reduced DREADD derivatives, therefore, has the potential to expand means in current brain sciences, where multimodality is required. In Figure S5, a cartoon discussing the possible advantage of delivering DREADDs with a single viral vector is available.

The availability of monkeys for basic research is limited due to ethical and legal constraints. In addition, monkeys exhibit considerable individual variability in both behavior and genetics. These features make it challenging to consistently experiment with multiple AAV vectors in different individuals compared to

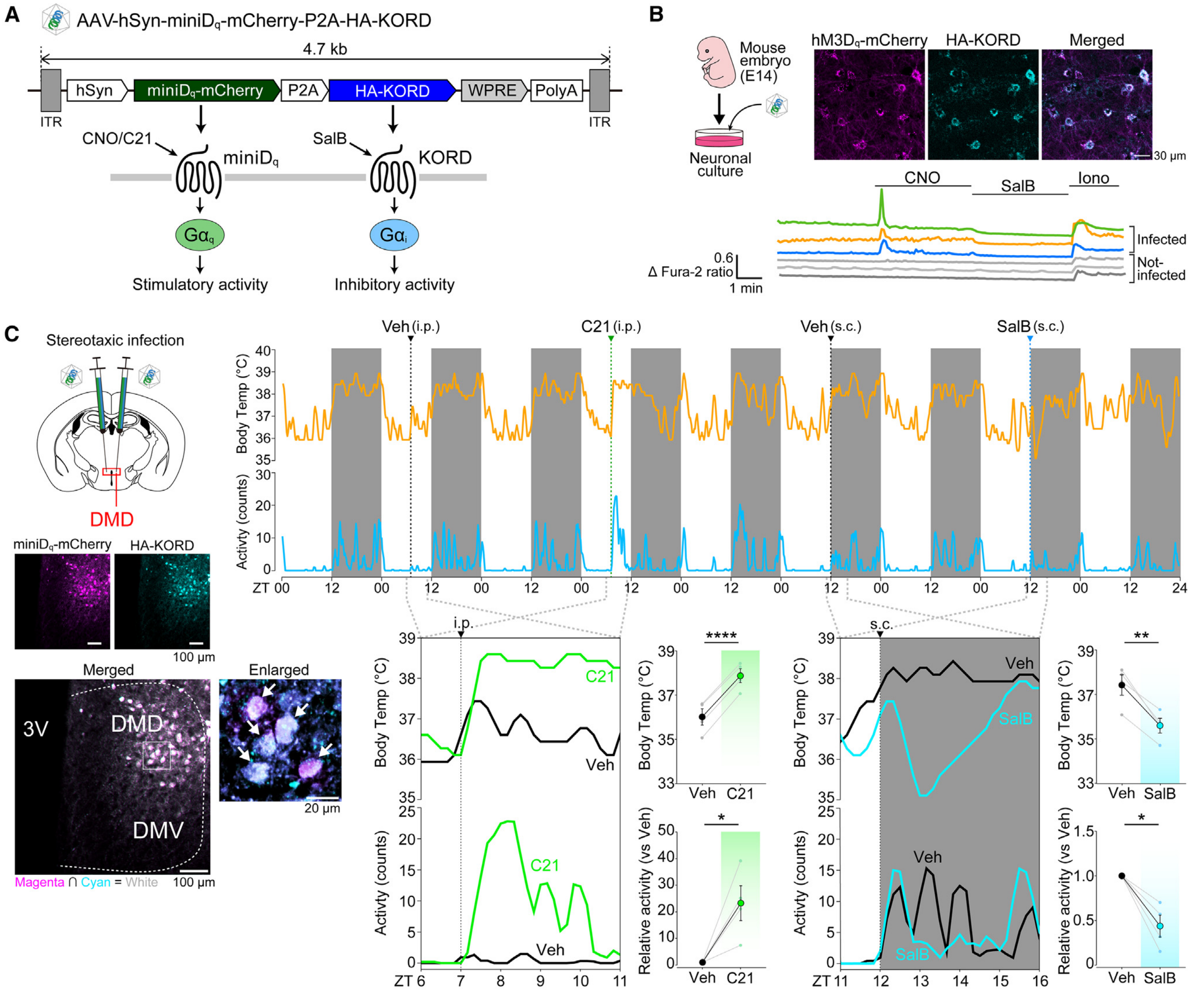

Figure 4. AAV-based DREADD system for bidirectional control of neuronal activity

(A) Schematic of the bidirectional DREADD system. AAV encodes both miniD$_q$ (neuronal activity enhancer) and KORD (repressor) within the limit of AAV packing capacity.

(B) Validation of the bidirectional DREADD system *in vitro*. Top, double-labeled confocal immunofluorescence of miniD$_q$-mCherry and HA-KORD in mouse primary neuronal cultures. Lower traces, representative Fura-2 ratio in AAV-infected (mCherry-positive) and not-infected cells. CNO, 100 nM; SalB, 50 μM.

(C) C21-induced upregulation and SalB-induced down-regulation of core body temperature and behavioral activity in mice virally expressing miniD$_q$ and KORD in DMD. A brain section verifies equivalent expression of miniD$_q$ (magenta) and KORD (cyan) within the same neuronal population in DMD (white, merged color). Data were analyzed using paired t test. Values are the mean ± SEM (n = 4 8-week-old male mice). ****$p < 0.0001$, **$p < 0.01$, and *$p < 0.05$.

studies using inbred mice. Our mini DREADD system may address (or at least mitigate) these issues by allowing the co-introduction and equivalent co-expression of excitatory and inhibitory DREADDs in the monkeys' brain. However, utilizing a single capsid simultaneously encoding miniD$_q$ and KORD, we were able to demonstrate consistent and bidirectional regulation of locomotor activity and body temperature in mice. This type of dual or sequential regulation can be achieved by using two separate viruses, albeit with a potentially variable infection efficiency between the two (Figure S5).[40–42] In addition, a single capsid method would contribute to reducing the virus titers available for use compared to double infection. This may help improve animal health by reducing unnecessary immune response in individual test animals.

In addition, our bidirectional AAV tool may instigate medical application consideration.[43] In recent years, a number of DREADD-based therapeutic approaches have been suggested for Parkinson disease,[44] Alzheimer disease,[45] depression,[46] and epilepsy.[38] Because the efficiency of AAV delivery varies among individuals, it is important to individualize the dosage of DREADD agonist. However, if only one excitatory or inhibitory DREADD is expressed, then there is no fail-safe option to rescue

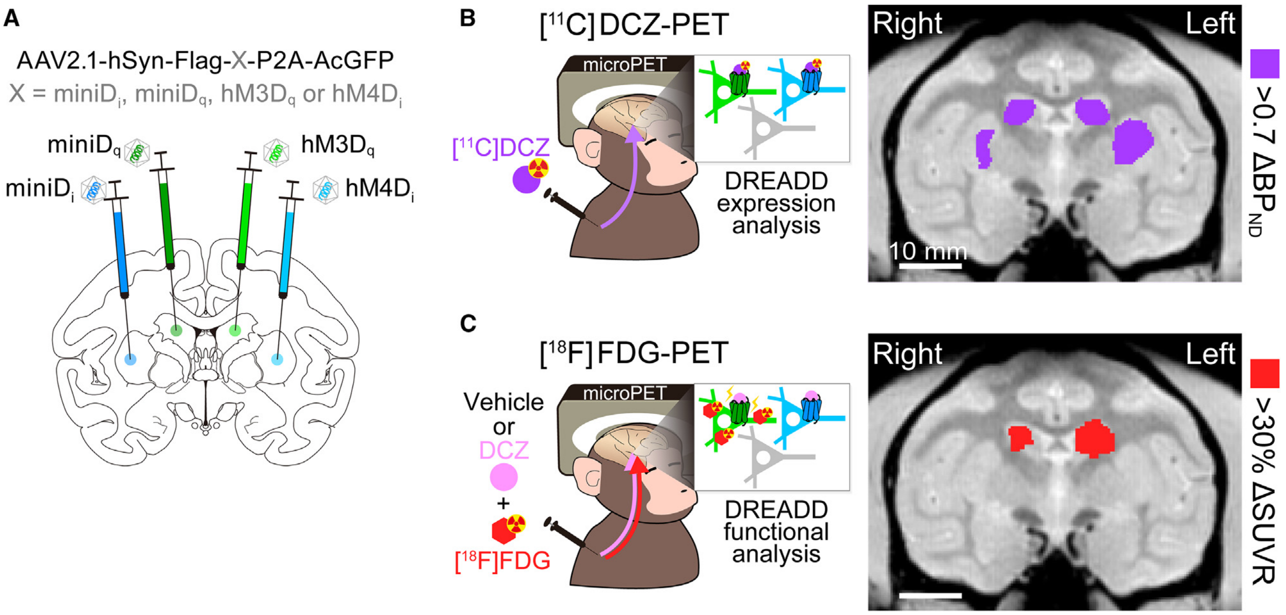

Figure 5. PET imaging of expression and function of mini DREADDs in monkeys

(A) Schematic illustration of striatal injection sites for $hM3D_q$, $miniD_q$, $hM4D_i$, and $miniD_i$.

(B) PET imaging of increased [^{11}C]DCZ binding after DREADD viral injection. A coronal image shows a significant increase in binding of [^{11}C]DCZ (purple; difference in binding potential, ΔBP_{ND} of >0.7) at the striatal injection sites, indicating successful expression of the receptors tested.

(C) PET imaging of [^{18}F]FDG uptake in the caudate nucleus following DCZ administration. The image shows an increase in [^{18}F]FDG uptake (≥30% of ΔSUVR, standardized uptake value relative to whole brain levels, compared with vehicle administration) specifically in regions of the caudate nucleus where $miniD_q$ and $hM3D_q$ were expressed.

inappropriately increased or down-regulated neuronal activity that could happen incidentally after drug application. In our system, both excitatory and inhibitory DREADDs are present in identical neurons at an equivalent fixed ratio, allowing sequential up- and down-regulation of neuronal activity, which may offer an opportunity to cancel excess activity by controlling the counteracting receptor activity. In addition, although the DREADD approach has not been previously considered as a potential treatment option for bipolar disorder, our tool might contribute to its revision since it can provide bidirectional neuronal control, a modality required for treating depressive and manic phases of this biphasic dysfunction.[47,48] Although purely hypothetical, our tool may end up offering homeostatic bidirectional control of neuronal activity in brain treatment.

The $miniD_q$ and $miniD_i$ plasmids that we made in this study have been deposited at the RIKEN BioResource Research Center (https://web.brc.riken.jp/) and the non-profit plasmid repository Addgene (http://www.addgene.org); all plasmids are publicly accessible. In the present study, we provided a series of experimental evidence showing that the basic receptor properties of $miniD_q$ and $miniD_i$ are conservative to those of the respective template receptors, $hM3D_q$ and $hM4D_i$, in terms of cell surface expression, ligand specificity and sensitivity, G-protein coupling subtype specificity, β-arrestin recruitment, and downstream ERK1/2 phosphorylation, while the agonist-independent basal activity observed at the ERK1/2 phosphorylation was slightly diminished in $miniD_i$ compared to that of the origin receptor $hM4D_i$. These lines of information would help promote the use of $miniD_i$/$miniD_q$ plasmids. Many of the previously reported modified receptors had high basal signaling *in vivo* that obscures ligand-induced phenotypes.[49,50] Thus, reduced basal activity, observed for $miniD_i$, is rather favorable for overexpressed chemogenetic tools. As a resource for researchers, the 30% size-reduced DREADD derivatives, with defined receptor characteristics, will expand the repertoire of receptors for conducting chemogenetic study.

Limitations of the study

In the current study, *in vivo* application of mini DREADDs was demonstrated using the mouse DMD and primate striatum as target sites. The applicability of mini DREADDs to other brain regions and their broader regulatory implications remain to be determined in future studies.

RESOURCE AVAILABILITY

Lead contact

Further information and requests for resources and reagents should be directed to and will be fulfilled by the lead contact, Masao Doi (doimasao@pharm.kyoto-u.ac.jp).

Materials availability

The $miniD_q$ and $miniD_i$ plasmids have been deposited to RIKEN BioResource Research Center (RBD no. 20117 for pAAV-hSyn-$miniD_q$-P2A-mCherry, no. 20118 for pAAV-hSyn-$miniD_i$-P2A-mCherry, and no. 20119 for pAAV-hSyn-$miniD_q$-mCherry-P2A-HA-KORD, https://web.brc.riken.jp/) and the non-profit plasmid repository Addgene (plasmids #204357 for pAAV-hSyn-$miniD_q$-P2A-mCherry, #204358 for pAAV-hSyn-$miniD_i$-P2A-mCherry, and #204359 for

pAAV-hSyn-miniD$_q$-mCherry-P2A-HA-KORD, http://www.addgene.org). All other reagents generated in this study are available from the lead contact with a completed materials transfer agreement.

Data and code availability

- Unprocessed original western blot (WB) and IHC data have been deposited at Mendeley Data and are publicly available at https://doi.org/10.17632/dj2x4748pc.1. All data reported in this paper will be shared by the lead contact upon request.
- This paper does not report original code.
- Any additional information required to reanalyze the data reported in this paper is available from the lead contact upon request.

ACKNOWLEDGMENTS

This work was supported in part by research grants from the Ministry of Education, Culture, Sports, Science and Technology of Japan (22H04987 to M.D., 24K02178 to T. Miyake, and 23H02405 to Y.N.), the Basis for Supporting Innovative Drug Discovery and Life Science Research program of the Japan Agency for Medical Research and Development (JP21am0101092), Kusunoki 125 of the Kyoto University 125th Anniversary Fund, the SRF, and the Astellas Foundation for Research on Metabolic Disorders (to M.D.), as well as the Moonshot R&D – MILLENNIA Program from the Japan Science and Technology Agency (JPMJMS2295) (to T. Minamimoto and K.-i.I.), the Kao Foundation for Arts and Sciences, the Uehara Memorial Foundation, SPIRIT2 2024 of Kyoto University, and ACT-X from the Japan Science and Technology Agency (JPMJAX222J) (to T. Miyake).

AUTHOR CONTRIBUTIONS

T. Miyake and M.D. conceived the project and designed the research; T. Miyake, K.T., and Y.I. contributed equally as first authors who performed experiments in collaboration with Y.N., R.N., T.S., S.N., K.-i.I., E.H., and T. Minamimoto; T. Miyake, T. Minamimoto, and M.D. wrote the paper with input from all authors; and M.D. supervised the project.

DECLARATION OF INTERESTS

The authors declare no competing interests.

STAR★METHODS

Detailed methods are provided in the online version of this paper and include the following:

- KEY RESOURCES TABLE
- EXPERIMENTAL MODEL AND STUDY PARTICIPANT DETAILS
  - Animals
  - Primary neuronal culture
  - Flp-In TREx293-DREADD cell lines
- METHOD DETAILS
  - Visualizing plasma membrane expression of DREADDs
  - Tango arrestin recruitment assay
  - Fura-2 Ca^{2+} imaging
  - GloSensor-cAMP assay
  - SRF-RE reporter assay
  - Immunoblotting
  - Viral preparation and infection
  - Locomotor activity and body temperature recording
  - PET imaging
- QUANTIFICATION AND STATISTICAL ANALYSIS

SUPPLEMENTAL INFORMATION

Supplemental information can be found online at https://doi.org/10.1016/j.crmeth.2024.100881.

Received: April 19, 2024
Revised: July 24, 2024
Accepted: September 23, 2024
Published: October 21, 2024

REFERENCES

1. Haggerty, D.L., Grecco, G.G., Reeves, K.C., and Atwood, B. (2020). Adeno-Associated Viral Vectors in Neuroscience Research. Mol. Ther. Methods Clin. Dev. *17*, 69–82. https://doi.org/10.1016/j.omtm.2019.11.012.
2. Challis, R.C., Ravindra Kumar, S., Chan, K.Y., Challis, C., Beadle, K., Jang, M.J., Kim, H.M., Rajendran, P.S., Tompkins, J.D., Shivkumar, K., et al. (2019). Systemic AAV vectors for widespread and targeted gene delivery in rodents. Nat. Protoc. *14*, 379–414. https://doi.org/10.1038/s41596-018-0097-3.
3. Goertsen, D., Flytzanis, N.C., Goeden, N., Chuapoco, M.R., Cummins, A., Chen, Y., Fan, Y., Zhang, Q., Sharma, J., Duan, Y., et al. (2022). AAV capsid variants with brain-wide transgene expression and decreased liver targeting after intravenous delivery in mouse and marmoset. Nat. Neurosci. *25*, 106–115. https://doi.org/10.1038/s41593-021-00969-4.
4. Halbert, C.L., Allen, J.M., and Miller, A.D. (2002). Efficient mouse airway transduction following recombination between AAV vectors carrying parts of a larger gene. Nat. Biotechnol. *20*, 697–701. https://doi.org/10.1038/nbt0702-697.
5. Kemaladewi, D.U., Maino, E., Hyatt, E., Hou, H., Ding, M., Place, K.M., Zhu, X., Bassi, P., Baghestani, Z., Deshwar, A.G., et al. (2017). Correction of a splicing defect in a mouse model of congenital muscular dystrophy type 1A using a homology-directed-repair-independent mechanism. Nat. Med. *23*, 984–989. https://doi.org/10.1038/nm.4367.
6. Tornabene, P., Trapani, I., Minopoli, R., Centrulo, M., Lupo, M., de Simone, S., Tiberi, P., Dell'Aquila, F., Marrocco, E., Iodice, C., et al. (2019). Intein-mediated protein trans-splicing expands adeno-associated virus transfer capacity in the retina. Sci. Transl. Med. *11*, eaav4523. https://doi.org/10.1126/scitranslmed.aav4523.
7. Duan, D., Goemans, N., Takeda, S., Mercuri, E., and Aartsma-Rus, A. (2021). Duchenne muscular dystrophy. Nat. Rev. Dis. Primers *7*, 13. https://doi.org/10.1038/s41572-021-00248-3.
8. Mok, Y.G., Lee, J.M., Chung, E., Lee, J., Lim, K., Cho, S.I., and Kim, J.S. (2022). Base editing in human cells with monomeric DddA-TALE fusion deaminases. Nat. Commun. *13*, 4038. https://doi.org/10.1038/s41467-022-31745-y.
9. Ran, F.A., Cong, L., Yan, W.X., Scott, D.A., Gootenberg, J.S., Kriz, A.J., Zetsche, B., Shalem, O., Wu, X., Makarova, K.S., et al. (2015). In vivo genome editing using Staphylococcus aureus Cas9. Nature *520*, 186–191. https://doi.org/10.1038/nature14299.
10. Li, A., Mitsunobu, H., Yoshioka, S., Suzuki, T., Kondo, A., and Nishida, K. (2022). Cytosine base editing systems with minimized off-target effect and molecular size. Nat. Commun. *13*, 4531. https://doi.org/10.1038/s41467-022-32157-8.
11. Rauch, S., He, E., Srienc, M., Zhou, H., Zhang, Z., and Dickinson, B.C. (2019). Programmable RNA-Guided RNA Effector Proteins Built from Human Parts. Cell *178*, 122–134.e12. https://doi.org/10.1016/j.cell.2019.05.049.
12. Xiong, M., Tao, Y., Gao, Q., Feng, B., Yan, W., Zhou, Y., Kotsonis, T.A., Yuan, T., You, Z., Wu, Z., et al. (2021). Human Stem Cell-Derived Neurons Repair Circuits and Restore Neural Function. Cell Stem Cell *28*, 112–126.e6. https://doi.org/10.1016/j.stem.2020.08.014.
13. Isberg, V., Mordalski, S., Munk, C., Rataj, K., Harpsøe, K., Hauser, A.S., Vroling, B., Bojarski, A.J., Vriend, G., and Gloriam, D.E. (2016). GPCRdb: an information system for G protein-coupled receptors. Nucleic Acids Res. *44*, D356–D364. https://doi.org/10.1093/nar/gkv1178.
14. Ichiyama, S., Oka, Y., Haga, K., Kojima, S., Tateishi, Y., Shirakawa, M., and Haga, T. (2006). The structure of the third intracellular loop of the

muscarinic acetylcholine receptor M2 subtype. FEBS Lett. *580*, 23–26. https://doi.org/10.1016/j.febslet.2005.11.042.

15. Doi, M., Murai, I., Kunisue, S., Setsu, G., Uchio, N., Tanaka, R., Kobayashi, S., Shimatani, H., Hayashi, H., Chao, H.W., et al. (2016). Gpr176 is a Gz-linked orphan G-protein-coupled receptor that sets the pace of circadian behaviour. Nat. Commun. *7*, 10583. https://doi.org/10.1038/ncomms10583.

16. Wang, T., Nakagawa, S., Miyake, T., Setsu, G., Kunisue, S., Goto, K., Hirasawa, A., Okamura, H., Yamaguchi, Y., and Doi, M. (2020). Identification and functional characterisation of N-linked glycosylation of the orphan G protein-coupled receptor Gpr176. Sci. Rep. *10*, 4429. https://doi.org/10.1038/s41598-020-61370-y.

17. Nakagawa, S., Nguyen Pham, K.T., Shao, X., and Doi, M. (2020). Time-Restricted G-Protein Signaling Pathways via GPR176, Gz, and RGS16 Set the Pace of the Master Circadian Clock in the Suprachiasmatic Nucleus. Int. J. Mol. Sci. *21*, 5055. https://doi.org/10.3390/ijms21145055.

18. Yamaguchi, Y., Murai, I., Takeda, M., Doi, S., Seta, T., Hanada, R., Kangawa, K., Okamura, H., Miyake, T., and Doi, M. (2022). Nmu/Nms/Gpr176 Triple-Deficient Mice Show Enhanced Light-Resetting of Circadian Locomotor Activity. Biol. Pharm. Bull. *45*, 1172–1179. https://doi.org/10.1248/bpb.b22-00260.

19. Kroeze, W.K., Sassano, M.F., Huang, X.P., Lansu, K., McCorvy, J.D., Giguère, P.M., Sciaky, N., and Roth, B.L. (2015). PRESTO-Tango as an open-source resource for interrogation of the druggable human GPCRome. Nat. Struct. Mol. Biol. *22*, 362–369. https://doi.org/10.1038/nsmb.3014.

20. Parikh, V., Kozak, R., Martinez, V., and Sarter, M. (2007). Prefrontal acetylcholine release controls cue detection on multiple timescales. Neuron *56*, 141–154. https://doi.org/10.1016/j.neuron.2007.08.025.

21. Armbruster, B.N., Li, X., Pausch, M.H., Herlitze, S., and Roth, B.L. (2007). Evolving the lock to fit the key to create a family of G protein-coupled receptors potently activated by an inert ligand. Proc. Natl. Acad. Sci. USA *104*, 5163–5168. https://doi.org/10.1073/pnas.0700293104.

22. Chen, X., Choo, H., Huang, X.P., Yang, X., Stone, O., Roth, B.L., and Jin, J. (2015). The first structure-activity relationship studies for designer receptors exclusively activated by designer drugs. ACS Chem. Neurosci. *6*, 476–484. https://doi.org/10.1021/cn500325v.

23. McDonough, R.C., Gilbert, R.M., Gleghorn, J.P., and Price, C. (2021). Targeted Gq-GPCR activation drives ER-dependent calcium oscillations in chondrocytes. Cell Calcium *94*, 102363. https://doi.org/10.1016/j.ceca.2021.102363.

24. Dhyani, V., Gare, S., Gupta, R.K., Swain, S., Venkatesh, K.V., and Giri, L. (2020). GPCR mediated control of calcium dynamics: A systems perspective. Cell. Signal. *74*, 109717. https://doi.org/10.1016/j.cellsig.2020.109717.

25. Goulding, J., May, L.T., and Hill, S.J. (2018). Characterisation of endogenous A_{2A} and A_{2B} receptor-mediated cyclic AMP responses in HEK 293 cells using the GloSensor^{TM} biosensor: Evidence for an allosteric mechanism of action for the A_{2B}-selective antagonist PSB 603. Biochem. Pharmacol. *147*, 55–66. https://doi.org/10.1016/j.bcp.2017.10.013.

26. Dates, A.N., Jones, D.T.D., Smith, J.S., Skiba, M.A., Rich, M.F., Burruss, M.M., Kruse, A.C., and Blacklow, S.C. (2024). Heterogeneity of tethered agonist signaling in adhesion G protein-coupled receptors. Cell Chem. Biol. *31*, 1542–1553.e4. https://doi.org/10.1016/j.chembiol.2024.03.004.

27. Qian, Y., Ma, Z., Liu, C., Li, X., Zhu, X., Wang, N., Xu, Z., Xia, R., Liang, J., Duan, Y., et al. (2022). Structural insights into adhesion GPCR ADGRL3 activation and G_q, G_s, G_i, and G_{12} coupling. Mol. Cell *82*, 4340–4352.e6. https://doi.org/10.1016/j.molcel.2022.10.009.

28. Labasque, M., Meffre, J., Carrat, G., Becamel, C., Bockaert, J., and Marin, P. (2010). Constitutive activity of serotonin 2C receptors at G protein-independent signaling: modulation by RNA editing and antidepressants. Mol. Pharmacol. *78*, 818–826. https://doi.org/10.1124/mol.110.066035.

29. Shenoy, S.K., Drake, M.T., Nelson, C.D., Houtz, D.A., Xiao, K., Madabushi, S., Reiter, E., Premont, R.T., Lichtarge, O., and Lefkowitz, R.J. (2006). beta-arrestin-dependent, G protein-independent ERK1/2 activation by the beta2 adrenergic receptor. J. Biol. Chem. *281*, 1261–1273. https://doi.org/10.1074/jbc.M506576200.

30. Zhao, Z.D., Yang, W.Z., Gao, C., Fu, X., Zhang, W., Zhou, Q., Chen, W., Ni, X., Lin, J.K., Yang, J., et al. (2017). A hypothalamic circuit that controls body temperature. Proc. Natl. Acad. Sci. USA *114*, 2042–2047. https://doi.org/10.1073/pnas.1616255114.

31. Gomez, J.L., Bonaventura, J., Lesniak, W., Mathews, W.B., Sysa-Shah, P., Rodriguez, L.A., Ellis, R.J., Richie, C.T., Harvey, B.K., Dannals, R.F., et al. (2017). Chemogenetics revealed: DREADD occupancy and activation via converted clozapine. Science *357*, 503–507. https://doi.org/10.1126/science.aan2475.

32. Hsiao, S.W., and Doi, M. (2024). Circuits involving the hypothalamic suprachiasmatic nucleus for controlling diverse physiologies verified by the aid of optogenetics and chemogenetics. Int. Rev. Cell Mol. Biol. https://doi.org/10.1016/bs.ircmb.2024.06.002.

33. Wu, Z., Yang, H., and Colosi, P. (2010). Effect of genome size on AAV vector packaging. Mol. Ther. *18*, 80–86. https://doi.org/10.1038/mt.2009.255.

34. Vardy, E., Robinson, J.E., Li, C., Olsen, R.H.J., DiBerto, J.F., Giguere, P.M., Sassano, F.M., Huang, X.P., Zhu, H., Urban, D.J., et al. (2015). A New DREADD Facilitates the Multiplexed Chemogenetic Interrogation of Behavior. Neuron *86*, 936–946. https://doi.org/10.1016/j.neuron.2015.03.065.

35. Nagai, Y., Miyakawa, N., Takuwa, H., Hori, Y., Oyama, K., Ji, B., Takahashi, M., Huang, X.P., Slocum, S.T., DiBerto, J.F., et al. (2020). Deschloroclozapine, a potent and selective chemogenetic actuator enables rapid neuronal and behavioral modulations in mice and monkeys. Nat. Neurosci. *23*, 1157–1167. https://doi.org/10.1038/s41593-020-0661-3.

36. Oyama, K., Hori, Y., Nagai, Y., Miyakawa, N., Mimura, K., Hirabayashi, T., Inoue, K., Suhara, T., Takada, M., Higuchi, M., and Minamimoto, T. (2021). Chemogenetic dissection of the primate prefronto-subcortical pathways for working memory and decision-making. Sci. Adv. *7*, eabg4246. https://doi.org/10.1126/sciadv.abg4246.

37. Hirabayashi, T., Nagai, Y., Hori, Y., Inoue, K., Aoki, I., Takada, M., Suhara, T., Higuchi, M., and Minamimoto, T. (2021). Chemogenetic sensory fMRI reveals behaviorally relevant bidirectional changes in primate somatosensory network. Neuron *109*, 3312–3322.e5. https://doi.org/10.1016/j.neuron.2021.08.032.

38. Miyakawa, N., Nagai, Y., Hori, Y., Mimura, K., Orihara, A., Oyama, K., Matsuo, T., Inoue, K., Suzuki, T., Hirabayashi, T., et al. (2023). Chemogenetic attenuation of cortical seizures in nonhuman primates. Nat. Commun. *14*, 971. https://doi.org/10.1038/s41467-023-36642-6.

39. Kimura, K., Nagai, Y., Hatanaka, G., Fang, Y., Tanabe, S., Zheng, A., Fujiwara, M., Nakano, M., Hori, Y., Takeuchi, R.F., et al. (2023). A mosaic adeno-associated virus vector as a versatile tool that exhibits high levels of transgene expression and neuron specificity in primate brain. Nat. Commun. *14*, 4762. https://doi.org/10.1038/s41467-023-40436-1.

40. Benekareddy, M., Stachniak, T.J., Bruns, A., Knoflach, F., von Kienlin, M., Künnecke, B., and Ghosh, A. (2018). Identification of a Corticohabenular Circuit Regulating Socially Directed Behavior. Biol. Psychiatry *83*, 607–617. https://doi.org/10.1016/j.biopsych.2017.10.032.

41. Dao, N.C., Brockway, D.F., Suresh Nair, M., Sicher, A.R., and Crowley, N.A. (2021). Somatostatin neurons control an alcohol binge drinking prelimbic microcircuit in mice. Neuropsychopharmacology *46*, 1906–1917. https://doi.org/10.1038/s41386-021-01050-1.

42. Suresh Nair, M., Dao, N.C., Lopez Melean, D., Griffith, K.R., Starnes, W.D., Moyer, J.B., Sicher, A.R., Brockway, D.F., Meeks, K.D., and Crowley, N.A. (2022). Somatostatin neurons in the bed nucleus of the stria terminalis play a sex-dependent role in binge Drinking. Brain Res. Bull. *186*, 38–46. https://doi.org/10.1016/j.brainresbull.2022.05.010.

CellPress
OPEN ACCESS

43. English, J.G., and Roth, B.L. (2015). Chemogenetics-A Transformational and Translational Platform. JAMA Neurol. *72*, 1361–1366. https://doi.org/10.1001/jamaneurol.2015.1921.

44. Alcacer, C., Andreoli, L., Sebastianutto, I., Jakobsson, J., Fieblinger, T., and Cenci, M.A. (2017). Chemogenetic stimulation of striatal projection neurons modulates responses to Parkinson's disease therapy. J. Clin. Invest. *127*, 720–734. https://doi.org/10.1172/JCI90132.

45. Rorabaugh, J.M., Chalermpalanupap, T., Botz-Zapp, C.A., Fu, V.M., Lembeck, N.A., Cohen, R.M., and Weinshenker, D. (2017). Chemogenetic locus coeruleus activation restores reversal learning in a rat model of Alzheimer's disease. Brain *140*, 3023–3038. https://doi.org/10.1093/brain/awx232.

46. Miller, O.H., Bruns, A., Ben Ammar, I., Mueggler, T., and Hall, B.J. (2017). Synaptic Regulation of a Thalamocortical Circuit Controls Depression-Related Behavior. Cell Rep. *20*, 1867–1880. https://doi.org/10.1016/j.celrep.2017.08.002.

47. Ashok, A.H., Marques, T.R., Jauhar, S., Nour, M.M., Goodwin, G.M., Young, A.H., and Howes, O.D. (2017). The dopamine hypothesis of bipolar affective disorder: the state of the art and implications for treatment. Mol. Psychiatry *22*, 666–679. https://doi.org/10.1038/mp.2017.16.

48. Vieta, E., Berk, M., Schulze, T.G., Carvalho, A.F., Suppes, T., Calabrese, J.R., Gao, K., Miskowiak, K.W., and Grande, I. (2018). Bipolar disorders. Nat. Rev. Dis. Primers *4*, 18008. https://doi.org/10.1038/nrdp.2018.8.

49. Guettier, J.-M., Gautam, D., Scarselli, M., Ruiz de Azua, I., Li, J.H., Rosemond, E., Ma, X., Gonzalez, F.J., Armbruster, B.N., Lu, H., et al. (2009). A chemical-genetic approach to study G protein regulation of beta cell function in vivo. Proc. Natl. Acad. Sci. USA *106*, 19197–19202. https://doi.org/10.1073/pnas.0906593106.

50. Saloman, J.L., Scheff, N.N., Snyder, L.M., Ross, S.E., Davis, B.M., and Gold, M.S. (2016). Gi-DREADD Expression in Peripheral Nerves Produces Ligand-Dependent Analgesia, as well as Ligand-Independent Functional Changes in Sensory Neurons. J. Neurosci. *36*, 10769–10781. https://doi.org/10.1523/JNEUROSCI.3480-15.2016.

51. Miyake, T., Nakamura, S., Zhao, M., So, K., Inoue, K., Numata, T., Takahashi, N., Shirakawa, H., Mori, Y., Nakagawa, T., and Kaneko, S. (2016). Cold sensitivity of TRPA1 is unveiled by the prolyl hydroxylation blockade-induced sensitization to ROS. Nat. Commun. *7*, 12840. https://doi.org/10.1038/ncomms12840.

52. Shao, X., Miyake, T., Inoue, Y., Hasegawa, E., and Doi, M. (2024). Temperature-Dependent Upregulation of Per2 Protein Expression Is Mediated by eIF2α Kinases PERK and PKR through PI3K Activation. Biol. Pharm. Bull. *47*, 600–605. https://doi.org/10.1248/bpb.b23-00739.

53. Fujita, Y., Miyake, T., Shao, X., Aoki, Y., Hasegawa, E., and Doi, M. (2024). Omeprazole Induces CYP3A4 mRNA Expression but Not CYP3A4 Protein Expression in HepaRG Cells. Biol. Pharm. Bull. *47*, 1218–1223. https://doi.org/10.1248/bpb.b24-00161.

54. Miyake, T., Inoue, Y., Shao, X., Seta, T., Aoki, Y., Nguyen Pham, K.T., Shichino, Y., Sasaki, J., Sasaki, T., Ikawa, M., et al. (2023). Minimal upstream open reading frame of Per2 mediates phase fitness of the circadian clock to day/night physiological body temperature rhythm. Cell Rep. *42*, 112157. https://doi.org/10.1016/j.celrep.2023.112157.

55. Nagai, Y., Kikuchi, E., Lerchner, W., Inoue, K., Ji, B., Eldridge, M.A.G., Kaneko, H., Kimura, Y., Oh-Nishi, A., Hori, Y., et al. (2016). PET imaging-guided chemogenetic silencing reveals a critical role of primate rostromedial caudate in reward evaluation. Nat. Commun. *7*, 13605. https://doi.org/10.1038/ncomms13605.

56. Doi, M., Shimatani, H., Atobe, Y., Murai, I., Hayashi, H., Takahashi, Y., Fustin, J.M., Yamaguchi, Y., Kiyonari, H., Koike, N., et al. (2019). Non-coding cis-element of Period2 is essential for maintaining organismal circadian behaviour and body temperature rhythmicity. Nat. Commun. *10*, 2563. https://doi.org/10.1038/s41467-019-10532-2.

57. Yan, X., Telu, S., Dick, R.M., Liow, J.S., Zanotti-Fregonara, P., Morse, C.L., Manly, L.S., Gladding, R.L., Shrestha, S., Lerchner, W., et al. (2021). [^{11}C]deschloroclozapine is an improved PET radioligand for quantifying a human muscarinic DREADD expressed in monkey brain. J. Cereb. Blood Flow Metab. *41*, 2571–2582. https://doi.org/10.1177/0271678X211007949.

STAR★METHODS

KEY RESOURCES TABLE

REAGENT or RESOURCE	SOURCE	IDENTIFIER
**Antibodies**		
anti-HA Alexa Fluor 647 conjugate	Cell Signaling	RRID: AB_10693329
anti-mCherry	Thermo Fisher	RRID: AB_2536611
anti-p44/42 MAPK	Cell Signaling	RRID: AB_330744
anti-phospho-p44/42 MAPK	Cell Signaling	RRID: AB_331646
anti-tubulin	Sigma	RRID: AB_477583
**Bacterial and virus strains**		
DH5α *Escherichia coli*	Takara Bio	#9057
**Chemicals, peptides, and recombinant proteins**		
Deschloroclozapine	MedChemExpress	HY-42110
Clozapine-N-oxide	Tocris	6329
Compound 21	Abcam	ab235545
Salvinorin B	Tocris	5611
[^{11}C]-deschloroclozapine	Nagai et al.[35]	N/A
[^{18}F]-fluoro-deoxy-glucose	Nagai et al.[35]	N/A
Acetylcholine chloride	Nacalai Tesque	00509–31
5′-N-ethylcarboxamido-adenosine	Tocris	1691
Thrombin	EMD Millipore	605190
Ionomycin	Nacalai Tesque	19444–91
Forskolin	Nacalai Tesque	16384–84
Pertussis toxin	BioAcademia	01–503
FR900359	Cayman	33666
Fura-2 a.m.	Dojindo	F015
**Deposited data**		
Unprocessed original WB and IHC data	This paper	Mendeley Data: https://doi.org/10.17632/dj2x4748pc.1
**Experimental models: cell lines**		
Flp-In TREx293	Thermo Fisher	R78007
**Experimental models: organisms/strains**		
C57BL/6J mice	Japan SLC	N/A
Cynomolgus monkey (Macaca fascicularis)	HAMRI Co., Ltd.	N/A
**Recombinant DNA**		
pAAV-hSyn-miniD$_q$-P2A-mCherry	This paper	RBD No. 20117; addgene #204357
pAAV-hSyn-miniD$_i$-P2A-mCherry	This paper	RBD No. 20118; addgene #204358
pAAV-hSyn-miniD$_q$-mCherry-P2A-HA-KORD	This paper	RBD No. 20119; addgene #204359
**Software and algorithms**		
ImageJ	NIH	https://imagej.nih.gov/ij/
Prism 8	GraphPad	N/A
Python 3.9	python	N/A

EXPERIMENTAL MODEL AND STUDY PARTICIPANT DETAILS

Animals

The C57BL/6J male mice 5–8 weeks old were purchased from Japan SLC (Shizuoka, Japan). We used only male mice in this study because the estrous cycle in females affects circadian rhythms of locomotor activity and body temperature. One macaque monkey

was used in the experiments (cynomolgus monkey; male, 3.9 kg, aged 3 years at the start of the experiments). All procedures for animal experiments were conducted in compliance with the Ethical Regulations of Kyoto University and the Guide for the Care and Use of Nonhuman Primates in Neuroscience Research (The Japan Neuroscience Society; https://www.jnss.org/en/animal_primates), were performed under protocols approved by the Animal Care and Experimentation Committee of Kyoto University and the Animal Ethics Committee of the National Institutes for Quantum Science and Technology, and were in accordance with the ARRIVE (Animal Research: Reporting of *In Vivo* Experiments) guidelines.

Primary neuronal culture

Primary neuronal cultures were prepared from the cortex of day 14 mouse embryos (E14). Cells were seeded on poly-D-lysine-coated coverslips and maintained in Neurobasal plus medium (Gibco) containing B27 plus supplement (Gibco) and penicillin/streptomycin/glutamine mixed solution. For immunostaining, cells were fixed, permeabilized, and blocked with 5% bovine serum albumin in PBS containing 0.1% Triton X-100, as described.[16] The cells were immunolabeled with anti-mCherry (Invitrogen, M11217) and visualized with Alexa 594-conjugated anti-rat IgG (Invitrogen, A-21209) and Alexa 647-conjugated anti-HA IgG (Cell Signaling, #3444). Images were captured using an Olympus FV10i-DOC confocal microscope.

Flp-In TREx293-DREADD cell lines

Flp-In TREx293-DREADD (tet-on)/GloSensor (constitutive) cells were generated by stable transfection of Flp-In TREx 293 cells (Thermo Fisher Scientific) with a modified pcDNA5/FRT vector encoding DREADD and GloSensor-22F (Promega) under different promoters: while DREADD was cloned into a proprietary pcDNA5/FRT cloning site for tet-on induction, *GloSensor* was cloned separately into a different position of the vector (at a unique *Pci*I site) in conjunction with a tet-insensitive CMV promoter. $G\alpha_{s/olf}$-deficient Flp-In TREx293 cells were generated using CRISPR/Cas9 genome editing technology. The sgRNA-encoding sequences targeting the *GNAS* ($G\alpha_s$) and *GNAL* ($G\alpha_{olf}$) were 5′-CTA CAA CAT GGT CAT CCG GG-3′ and 5′-GTA ATG TTT GCC GTC ACC GG-3′, respectively, both cloned in the pSpCas9 (BB)-2A-Puro vector. Cells were cultured at 37°C under 5% CO_2 in DMEM medium (Nacalai) containing 10% fetal bovine serum and required antibiotics according to the manufacture's protocol as described previously.[16] To induce DREADD expression, doxycycline (Dox, Clontech) was added to the medium to a final concentration of 1 μg/mL.

METHOD DETAILS

Visualizing plasma membrane expression of DREADDs

We constructed mCherry fusion expression vectors for $miniD_q$, $miniD_i$, $hM3D_q$ and $hM4D_i$. The receptors were C-terminally fused with mCherry. HEK293 cells were transfected with the DREADD-mCherry vectors using Viofectin Transfection Reagent (Viogene). We visualized subcellular location of mCherry-derived fluorescence in cells plated on poly-D-lysine-coated glass-bottom dish. Confocal images were obtained using an Olympus FV10i-DOC microscope (Olympus).

Tango arrestin recruitment assay

DREADDs were cloned into the PREST-Tango expression vector (Addgene #66227). The PREST-Tango β-arrestin recruitment assay was performed as described[19] except adopting transient transfection of a DREADD-Tango of interest and TRE-Luc2CP-CMV-β-arrestin2-TEV expression vector to HEK293 cells. For the co-transfection of β-arrestin2-TEV and TRE-Luc2CP, the Luc2CP sequence (Promega) under TRE3G promotor (Takara) was cloned into a unique *Mfe*I site of pcDNA3.1-bArrestin2-TEV vector (Addgene #107245). Cells were treated with different concentrations of compound 21 (C21) or deschloroclozapine (DCZ) in the presence of 1 mM luciferin (Promega). Luminescence was measured using a dish-type luminometer (Kronos-Dio, ATTO) maintained at 35°C. Recording was performed for 2 min for each dish at 30-min intervals. The obtained values were normalized to the maximum response of C21 or DCZ set at 100% and the concentration-response curves were fitted in GraphPad Prism 8.

Fura-2 Ca^{2+} imaging

Cells were preincubated with 5 μM Fura-2 a.m.-containing Krebs-Ringer solution for 30 min before experiment. Fluorescence images (excitation at 340 or 380 nm and emission at 510 nm) were captured every 5 s at room temperature as described.[51] The ratio of F340 to F380 was used as a relative indicator for intracellular Ca^{2+} concentration. The mean values at 4–5 min post C21 or DCZ treatment were determined for >80 cells in each experiment. For detecting Ca^{2+} in mouse primary neuronal cells, we treated cells with CNQX to reduce confounding signals from excitatory glutamatergic transmission (Sigma, 5 μM).

GloSensor-cAMP assay

Flp-In TREx293-DREADD (tet-on)/GloSensor (constitutive) cells were seeded in a collagen I-coated 96-well plate (Corning) at a density of 5.4×10^4 cells per well with a carbon dioxide-independent DMEM (Sigma, D2902) containing 10% bovine serum, 0.035% $NaHCO_3$, 10 mM HEPES (pH 7.2), 3.5 g/L D-glucose, 1% Antibiotic-Antimycotic Mixed solution (Nacalai), and 1 mM luciferin (Promega). After 6 h at 37°C, the cells received Dox or vehicle and underwent additional incubation at 37°C for >15 h. Prior to luminescence detection, the cell culture plate was acclimatized to 27°C for 1 h. Luminescence was then recorded on an FDSS/μCELL plate reader

(Hamamatsu Photonics) at 27°C every 5 s. Data were integrated over 1-min intervals, and the values were normalized to the average of C21 (−), DCZ (−) or Dox (−) cells. To monitor G_i activity, forskolin (Fsk, 10 μM, Nacalai) was applied.

SRF-RE reporter assay

We generated 5×SRF-RE-Luc2CP expression vector by inserting five SRF-responsive elements (5′-ATG TCC ATA TTA GGA CAT CT-3′) into *Kpn*I-*Hin*dIII sites of pGL4.25 reporter plasmid. The $G\alpha_{s/olf}$-deficient Flp-In TREx293 cells were transiently transfected with the pcDNA3 vector containing either $hM3D_q$, $hM4D_i$, $miniD_q$, or $miniD_i$ together with the pGL4.25-5×SRF-RE-Luc2CP vector and were cultured for 24 h. Then, the cells were treated with pertussis toxin (PTX, 100 ng/mL, BioAcademia) and FR900359 (FR, 0.5 μM, Cayman) for 3 h prior to stimulation with C21 (100 nM) or DCZ (1 nM). As a control, pcDNA3 vector encoding PAR1 was transfected and its response to thrombin (Thr, 3 U, EMD Millipore) was monitored in parallel. Luminescence was measured at 37°C using Kronos-Dio, as described previously.[52] Recordings were performed for 2 min at 30-min intervals. The obtained values were normalized to the average of C21 (−), DCZ (−) or Thr (−) cells.

Immunoblotting

For sample preparation, cells were directly lysed into Laemmli buffer containing 1x cOmplete Protease Inhibitor Cocktail (Roche) after treatment with C21(final concentration, 1 μM) or DCZ (100 nM) or Dox (1 μg/mL) in culture. Immunoblotting was performed as described[53] using commercially available antibodies against α-tubulin (Sigma, T6199, 1:1,000), p44/42 MAPK (Cell signaling, #9102, 1:1,000), or phospho-p44/42 MAPK (Cell signaling, #9101, 1:1,000).

Viral preparation and infection

AAV-DREADD was produced using a triple-transfection, helper-free method as described.[54] Purified AAV, whose titer was >1.0× 10^{13} genome copies per milliliter, was then injected into animals. Under anesthesia, mice received bilateral stereotaxic injections of AAV, 0.5 μL per site, into the DMD (at −1.25-mm posterior, ±0.3-mm lateral, −5.0-mm ventral, relative to the bregma). Behavioral studies were performed 3–4 weeks after the injection. To infect mouse primary neuronal cells, cells were incubated in AAV-containing culture medium for >14 days to achieve sufficient gene expression.

For viral injections into monkey brain, anesthesia was induced using intramuscular (i.m.) injection of ketamine (5–10 mg/kg) and xylazine (0.2–0.5 mg/kg), and maintained with isoflurane (1%–3%, to effect). AAV vectors (2 μL per site) were pressure-injected into the striatum using a 10-μL microsyringe (Model 1701RN, Hamilton) with a 30-gauge injection needle placed in a fused silica capillary (450 μm OD), which minimizes backflow by creating a 500-μm space surrounding the needle tip.[55] The injection rate was set at 0.2 μL/min. Stereotaxic coordinates of the injected sites were determined from overlaid magnetic resonance (MR) and computed tomography (CT) images created by PMOD image analysis software (PMOD Technologies, Zurich, Switzerland).[39]

Locomotor activity and body temperature recording

We used adult male C57BL/6J mice (8–12 weeks old) housed individually in light-tight, ventilated closets under indicated lighting conditions with *ad libitum* access to food and water. Locomotor activity was recorded via passive infrared sensors (PIRs, FA-05F5B; Omron) with 1-min resolution and analyzed with CLOCKLAB software (Actimetrics). Body temperature was recorded using precalibrated temperature data loggers (Thermochron iButtons, DS1921H, Maxim) implanted into the peritoneal cavity of mice as described.[56] Where indicated, mice received clozapine-N-oxide (CNO, 3 mg/kg, i.p.), C21 (1 mg/kg, i.p.), DCZ (100 μg/kg, i.p.), or salvinorin B (SalB, 5 mg/kg, s.c.). Successful expression of the virus was confirmed by immunohistochemistry after the recordings. For the calculation, body temperature at 1.5 h after drug application and mean locomotor activity values at 0.5–3 h after drug application were used to see the effects of drugs. Expression of $miniD_q$ and KORD in the DMD was confirmed by post hoc immuno-histochemistry by using rat anti-mCherry IgG (Invitrogen, M11217), Alexa 594-conjugated anti-rat IgG (Invitrogen, A-21209) and Alexa 647-conjugated anti-HA IgG (Cell Signaling, #3444).

PET imaging

PET imaging was performed using the procedures described previously.[35] Briefly, the monkey was sedated with ketamine hydrochloride (5 mg/kg, i.m.) and xylazine hydrochloride (0.5 mg/kg, i.m.), and the anesthetized condition was maintained with isoflurane (1–2%, inhalation) during the PET imaging. PET scans were performed with a microPET Focus220 scanner (Siemens Medical Solutions). Following transmission scans, emission scans were acquired for 90 min after intravenous bolus injection of [^{11}C]DCZ (323.7–358.2 MBq) or [^{18}F]FDG (196.5–226.0 MBq). Pretreatment with DCZ (5 μg/kg) or vehicle (1–2% DMSO in 0.1-mL saline, without DCZ) was carried out 1 min before the [^{18}F]FDG injection. The PET imaging data were reconstructed with filtered back-projection with attenuation correction. Voxel values were converted to standardized uptake values (SUVs) that were normalized by injected radioactivity and body weight using PMOD (PMOD Technologies). Volumes of interest (VOIs) were manually drawn on the center of the injection site and the cerebellum using PMOD, by referring to MR images of individual monkeys. To estimate the specific binding of [^{11}C]DCZ, the regional binding potential relative to non-displaceable radioligand (BP_{ND}) was calculated with an original multilinear reference tissue model using the cerebellum as a reference region.[57] For FDG-PET analysis, dynamic SUV images were motion-corrected and then averaged between 30 and 60 min after the radioligand injection. The SUV ratio (SUVR) of voxel value was calculated as a percentage of the averaged value of the whole brain for comparison between the scans.

QUANTIFICATION AND STATISTICAL ANALYSIS

Western blot band intensities were quantified using ImageJ software. Statistical analyses and plots were generated with GraphPad Prism 8 and Python 3.9, using the statistical tests indicated in the figure legends.

Cell Reports Methods, Volume *4*

Supplemental information

Size-reduced DREADD derivatives for AAV-assisted multimodal chemogenetic control of neuronal activity and behavior

Takahito Miyake, Kaho Tanaka, Yutsuki Inoue, Yuji Nagai, Reo Nishimura, Takehito Seta, Shumpei Nakagawa, Ken-ichi Inoue, Emi Hasegawa, Takafumi Minamimoto, and Masao Doi

<u>**Supplementary Figure 1**</u>

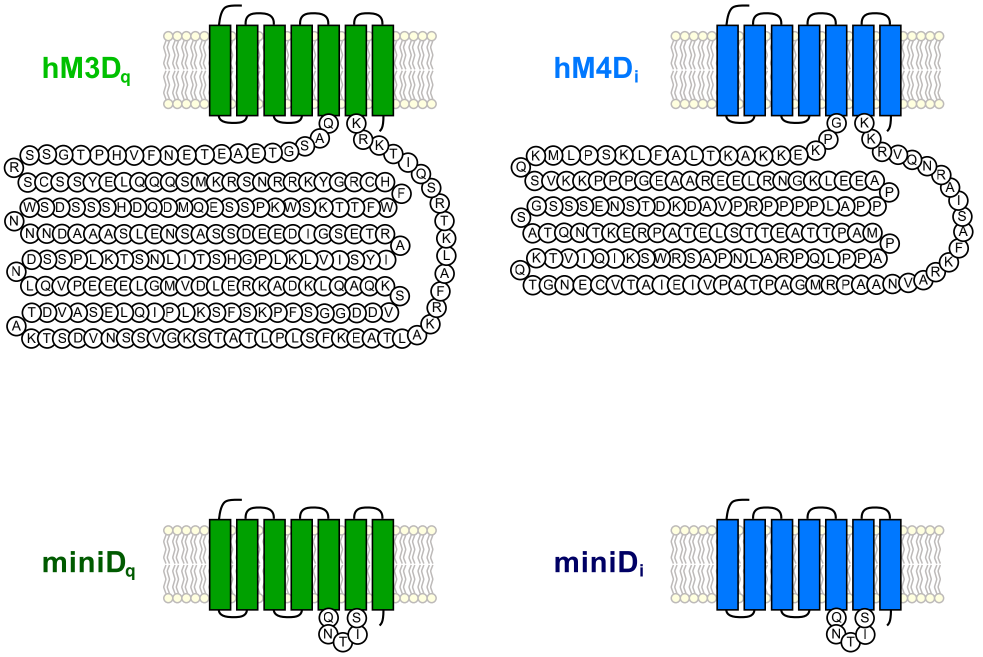

Supplementary Figure 1 (related to Fig. 1) | Snake plots showing the amino acid sequence of the ICL3 region of hM3D$_q$, hM4D$_i$, miniD$_q$ and miniD$_i$. We substituted the ICL3 of hM3D$_q$ and hM4D$_i$ with a 5-amino-acid peptide sequence Q-N-T-I-S.

Supplementary Figure 2

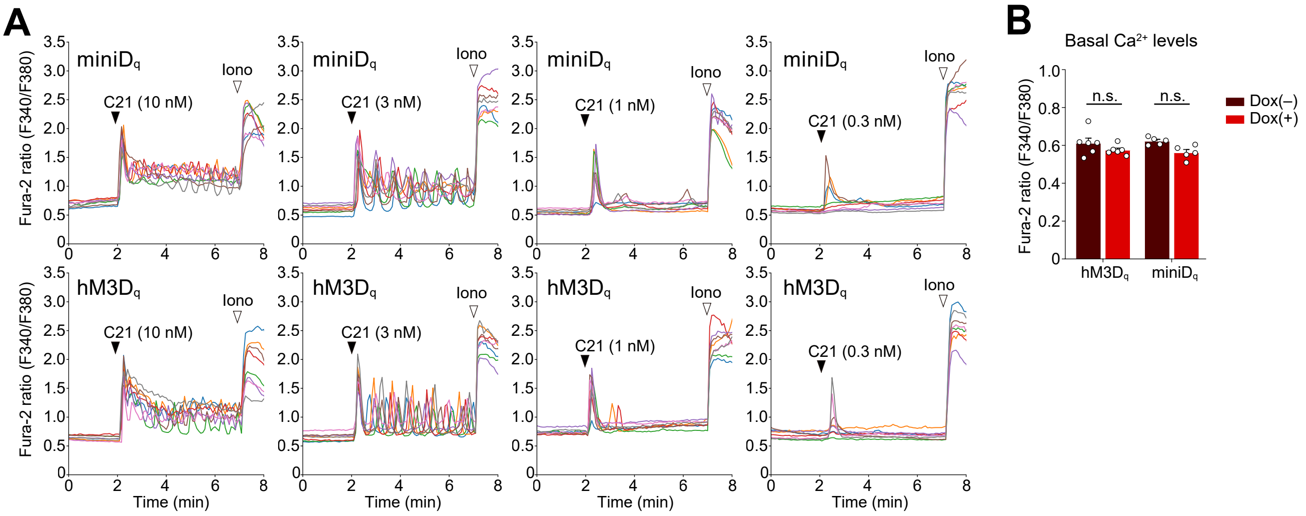

Supplementary Figure 2 (related to Fig. 2) | Fura-2 Ca^{2+} imaging of D_q-DREADD- expressing Flp-In TREx293 cells. (A) Representative traces in D_q-DREADD expressing cells. The traces of 10 nM C21-treated are the same as Figure 2D. Iono, ionomycin (3 μM). C21 evoked an immediate increase in Ca^{2+}, which is followed by oscillatory fluctuations of Ca^{2+}. Note that Ca^{2+} fluctuations after 3 nM C21 treatment were obvious and similar between hM3D_q and hM3D_q-ICL3$_{176}$ (miniD_q). **(B)** Comparison of basal Ca^{2+} levels between Dox-treated and -untreated cells for hM3D_q and miniD_q. *n* = 5−6 biological replicates. Cells were pretreated with Dox or vehicle for 18 h and monitored for $[Ca^{2+}]_i$ without C21 administration. Values are the means ± SEM. Two-way ANOVA followed by Sidak's multiple comparisons test. n.s., not significant.

Supplementary Figure 3

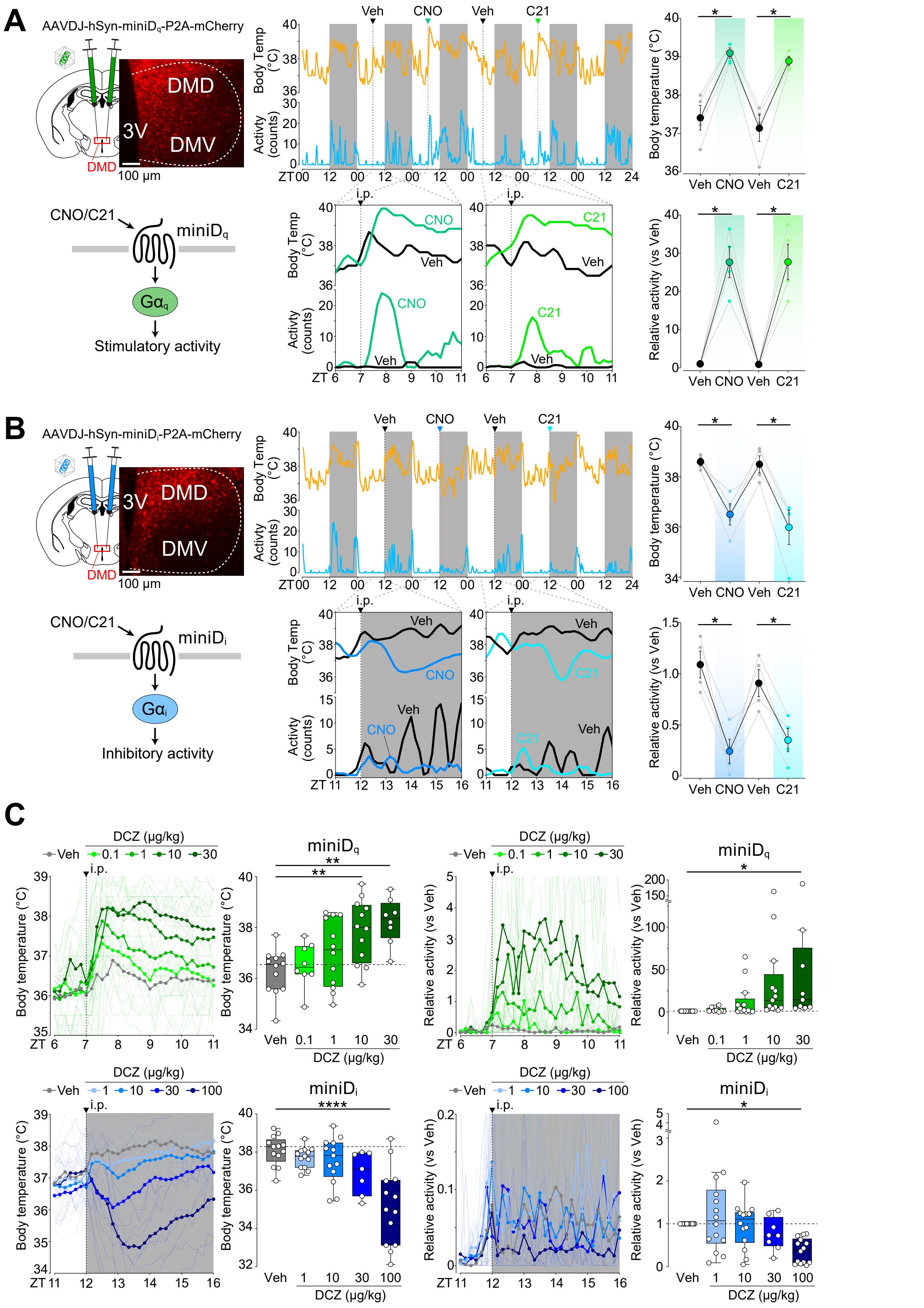

Supplementary Figure 3 (related to Fig. 4) | In vivo functionality of miniD$_i$/miniD$_q$-mediated neuronal activation or inhibition. (A) C21/CNO-induced upregulation of core body temperature and behavioral activity in mice virally expressing miniD$_q$ in the DMD. *n* = 4 mice. **(B)** C21/CNO-induced downregulation of core body temperature and behavioral activity in mice virally expressing miniD$_i$ in the DMD. *n* = 4 mice. Values in (A) and (B) are the mean ± SEM. **(C)** DCZ dose-dependent regulation of core body temperature and behavioral activity in mice virally expressing miniD$_q$ or miniD$_i$ in the DMD. *n* = 7–14 mice. In all experiments, we used male mice of 8-week-old. Mice were i.p. injected with DCZ (0.1–100 μg/kg), CNO (3 mg/kg), C21 (1 mg/kg), or vehicle (Veh, saline). *p < 0.05; **p < 0.01; ****p < 0.0001, one-way ANOVA followed by Tukey (A, B) or Dunnett (C) multiple comparisons test.

Supplementary Figure 4

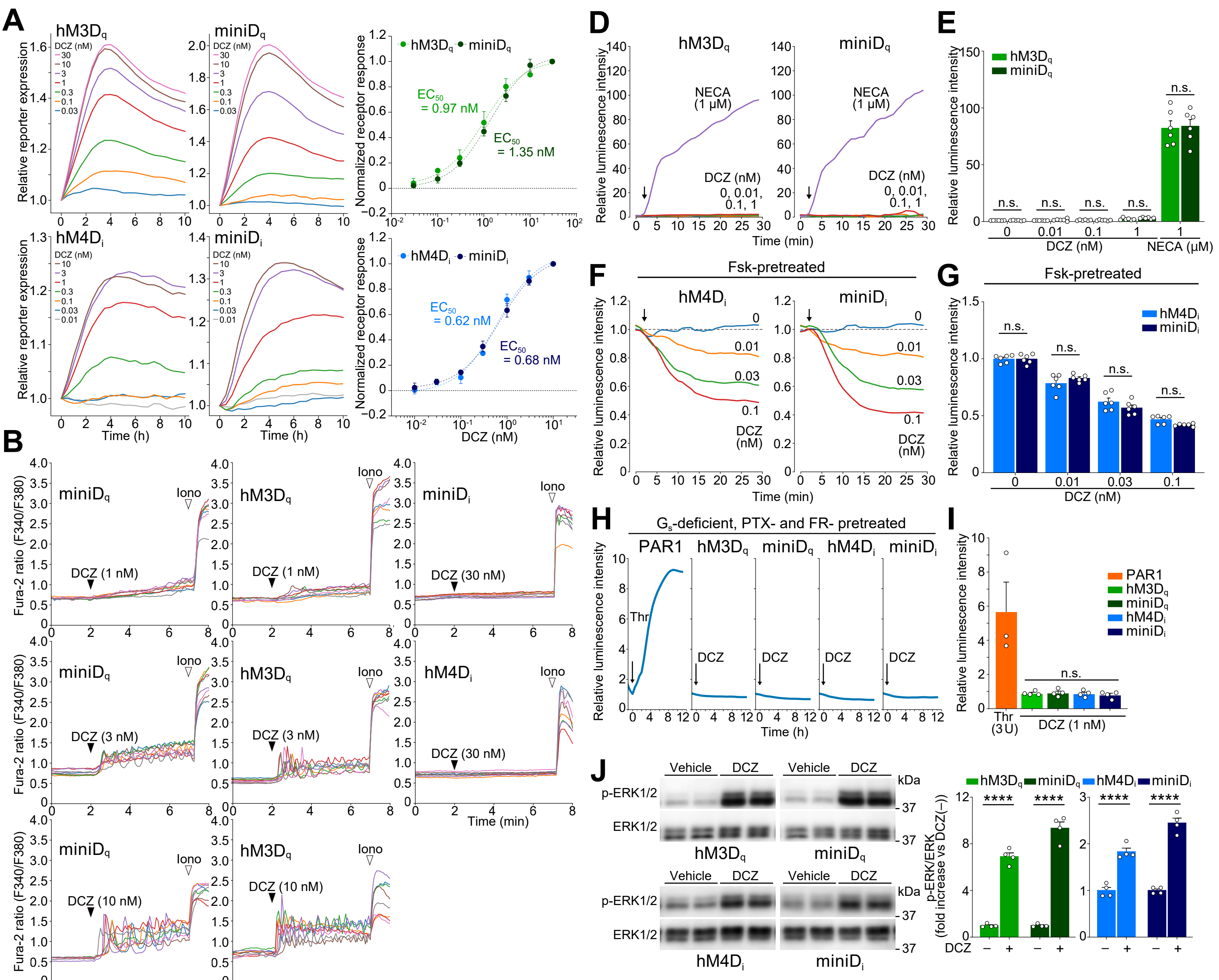

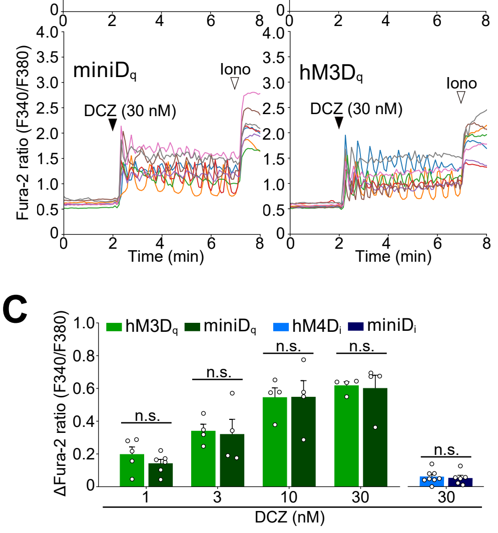

Supplementary Figure 4 (related to Fig. 5) | Conserved DCZ sensitivity in mini DREADDs in vitro. (A) Representative traces (left) and dose-response curves (right) of Tango reporter activity for hM3D$_q$, miniD$_q$, hM4D$_i$ and miniD$_i$ in response to DCZ treatment in HEK293 cells. n = 3−5 biological replicates. **(B)** Representative Fura-2 ratio traces in Flp-In TREx293 cells expressing hM3D$_q$, miniD$_q$, hM4D$_i$ or miniD$_i$. Arrowheads at 2 min represent the start of DCZ treatment. **(C)** Quantification of sustained Ca^{2+} levels in each DREADD receptor-expressing cells after treatment of increasing doses of DCZ. n = 4−8 biologically independent experiments. **(D–G)** Representative DCZ-induced changes in cAMP GloSensor reporter activity and their statistical quantification for hM3D$_q$ and miniD$_q$ (D, E) and hM4D$_i$ and miniD$_i$ (F, G). n = 6 biological replicates. **(H, I)** Representative SRF-RE reporter activity traces (H) and their statistical quantification (I). n = 3–4 biological replicates. Arrows, drug application. **(J)** Immunoblotting and densitometric analysis showing a significant and comparable increase in ERK phosphorylation after DCZ treatment in cells expressing hM3D$_q$, miniD$_q$, hM4D$_i$ and miniD$_i$. n = 4 biological replicates. Data were analyzed using two-way ANOVA followed by Sidak's multiple comparisons test (E, G, J, and hM3D$_q$ vs miniD$_q$ in C), unpaired Student's *t*-test (hM4D$_i$ vs miniD$_i$ in C) or one-way ANOVA followed by Tukey's multiple comaprisons test (I). Values are the means ± SEM. ****$p < 0.0001$; n.s., not significant.

Supplementary Figure 5

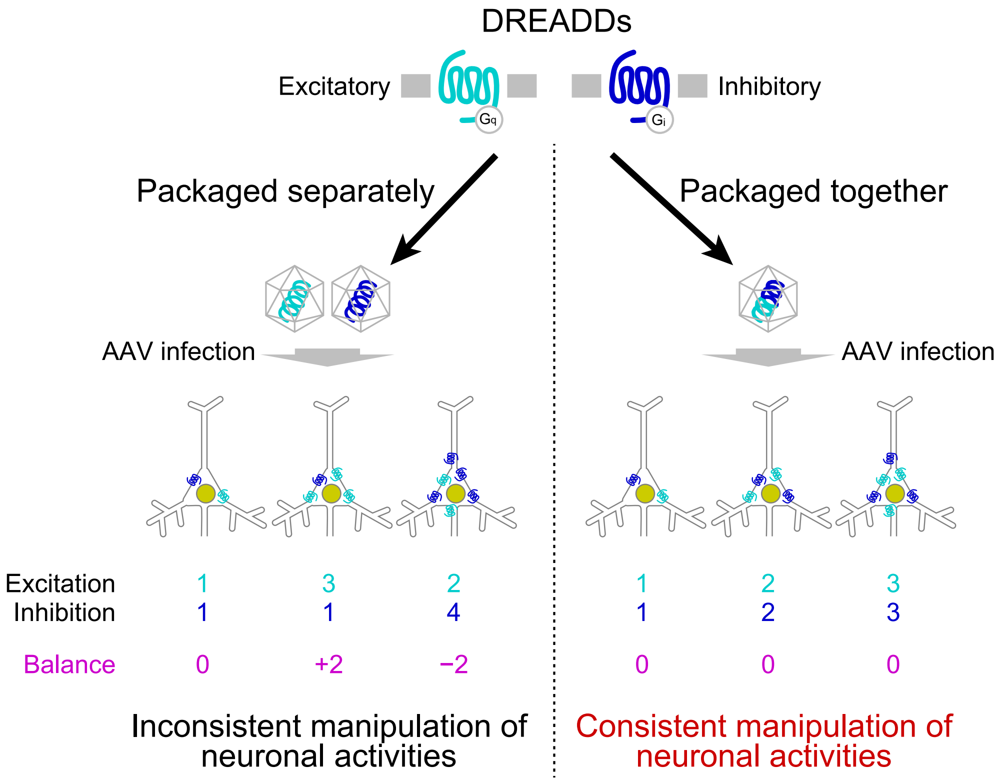

Supplementary Figure 5 (related to Figs. 4 and 5) | Schematic illustrating the potential advantage of co-packaging excitatory and inhibitory DREADDs in a single AAV capsid. When the two DREADDs are packaged separately in different AAV vectors, the ratio of excitatory to inhibitory DREADD expression can vary across neurons, resulting in inconsistent neuronal manipulation (left). In contrast, co-packaging the two DREADDs within a single capsid ensures the co-introduction of the two receptors to the same neurons with expression ratios equivalent between the two receptors, which would allow consistent up/down manipulation of targeted neuronal activities (right).
